# Investigating intentional cranial modification: A hybridized two-dimensional/three-dimensional study of the Hirota site, Tanegashima, Japan

**DOI:** 10.1371/journal.pone.0289219

**Published:** 2023-08-16

**Authors:** Noriko Seguchi, James Frances Loftus, Shiori Yonemoto, Mary-Margaret Murphy

**Affiliations:** 1 Faculty of Social and Cultural Studies, Kyushu University, Fukuoka, Japan; 2 Department of Anthropology, The University of Montana, Missoula, MT, United States of America; 3 Japan Society for the Promotion of Science (JSPS), Tokyo, Japan; 4 The Kyushu University Museum, Kyushu University, Fukuoka, Japan; University of California Santa Cruz, UNITED STATES

## Abstract

Intentional cranial modification has a long history, being a ubiquitous practice in many cultures around the world for millennia. The crania excavated at the Hirota site on Tanegashima Island, Kagoshima Prefecture, Japan, has been previously noted to have a marked tendency toward a short head and a flattened occipital bone, which has been suggested to be the result of artificial cranial deformation. However, whether this deformation was intentional or caused by unintentional habits remains unclear. This study aimed to investigate the characteristics of the cranial shape of the Hirota site to clarify whether the crania were intentionally modified. In the examination of Hirota crania, Kyushu Island Jomon and Doigahama Yayoi crania were added as comparative data and contrasted with three-dimensional (3D) surface scan imaging and two-dimensional outline-based geometric morphometric analysis, combined with objective assessments of potential cranial modification. The results showcased Hirota’s short and flattened cranial morphology, indicating clear alignment with our hypothesis that Hirota samples are morphologically different from Doigahama and Jomon samples. No sex-based differences were found. Morphological abnormalities in cranial sutures were visually assessed utilizing novel 3D visualization methods of cranial outer surfaces. Based on a comprehensive review of the results, we concluded that Hirota site crania were intentionally modified. Although the motivation of the practice is unclear, the Hirota people may have deformed their crania to preserve group identity and possibly aid in the long-distance trade of shellfish, as seen archaeologically.

## Introduction

Intentional cranial modification (ICM) has been a continuously ubiquitous practice in various cultures on all continents. Archaeological evidence has shown that humans have permanently altered the appearance of their heads since antiquity [1–8]. In China, a possible intentionally modified cranium was observed in at least one individual (UC 102) among three crania from the Upper Cave of the Zhoukoudian [4, 9, 10]. In Australia, many crania from Kow Swamp, Coobool Creek, and Nacurrie also exhibit ICM [2–5, 11]. Those modified crania display a high frequency of hyperostotic traits, such as excessive growth or thickening of bone tissues [1, 3–5]; however, humans have continuously practiced ICM, as head-splinting, binding practices, and cradling traditions have continuously evolved across the globe for large swaths of time [12]. Furthermore, the Neolithic Houtaomuga site in Northern China is the earliest confirmed case of ICM from the easternmost Old World, where ICM was practiced ranging from 12,000 to 5,000 BP [13].

In the Andes and pre-Columbian Mesoamerica, permanent body modification such as ICM is well studied and explored with rich excavated archaeological artifacts. Such studies address body modification from not only biological and physiological, but also sociocultural aspects, incorporating sociocultural, agency, and body theories, such as mind and body and embodiment [12]. Based on bioarchaeological evidence, researchers interpret head-shaping as part of the formation of a group identity, which includes symbols of social status, cultural belongings, beauty, ideology, beliefs, and gender [12–15]. Tiesler [12] sheds light on differential gender-based practices in ancient societies and argues that ICM could have been performed daily by female caretakers and mothers on their infant kin; s indicating this practice was an aspect of vertical social learning from generation to generation and old to young. Tiesler [12, 16] also examined the female craft of head shapes and their role in family succession and group identity, and explored the structural relationships established with society by the individual. Elaborating on these discussions, many social and cultural aspects of ICM-practicing societies could be enlightened beyond just the osteological and biological consequences of the practice of ICM.

In Japan, possible ICM in the Yoshigo site Jomon period population (ca. 3200–2800 cal BP) on the Atsumi Peninsula of Tahara city, Aichi Prefecture, was reported by Kiyono and Hirai [17] and by Kanataka and Kanaseki [18]. Kanaseki and Ogata [19] also reported a possible deformed cranium at the Koura site from the Yayoi period (1900–1700 BP). According to them, this cranium displayed circular depressions on the midline of the frontal bone; however, no visually observable modified cranial shapes were seen beyond these frontal depressions, and thus, may have been unintentional traces left by the habitual practice of circular banding. Regardless, such potential banding practices shed light on potential cases of banding during the Yayoi period on the Japanese archipelago.

**Fig 1:**
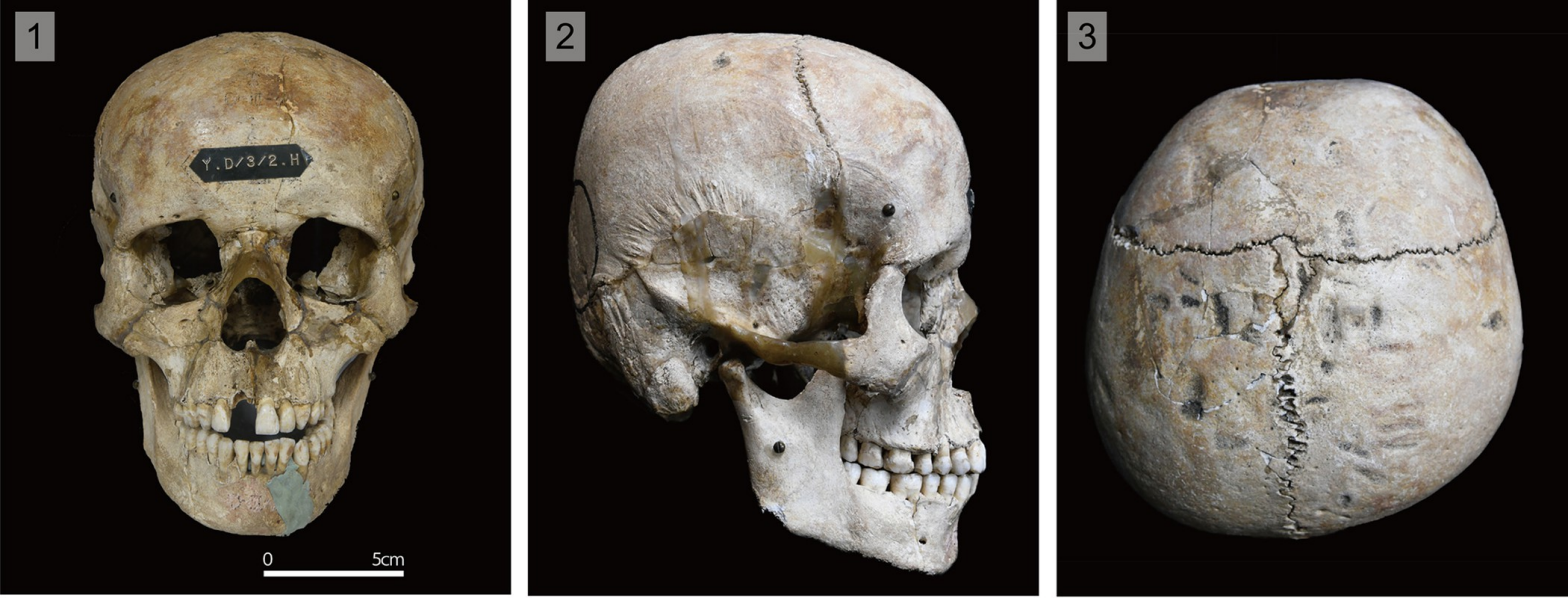
Individual ‘HT4’ Hirota site crania. 1) Frontal View; 2) Right-lateral view; 3) Superior view.

**Fig 2:**
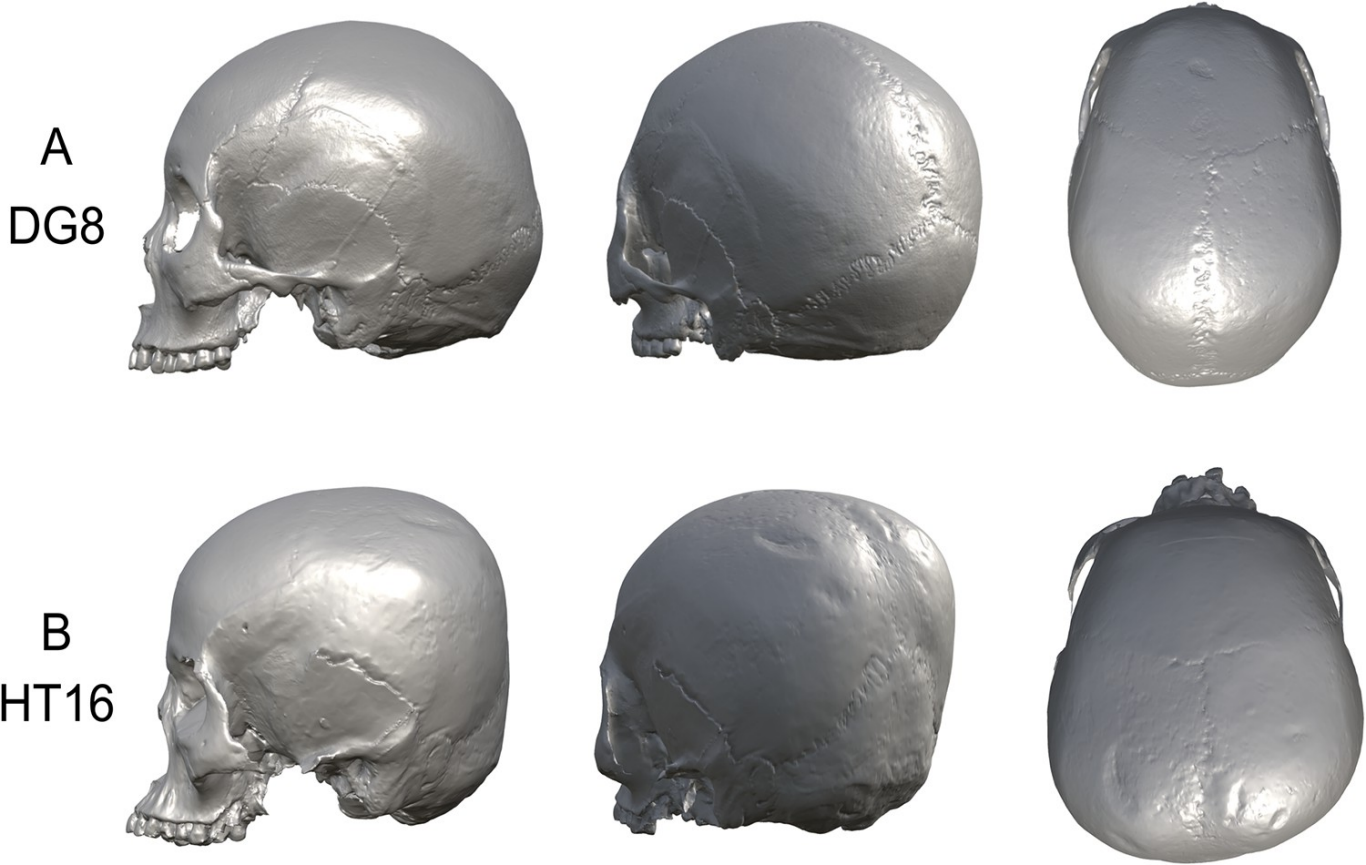
Morphological comparison of Doigahama and Hirota example crania. A: Doigahama sample DG8; B: Hirota sample HT16. HT16 shows an extreme deformation of the posterior region resulting in a flattened and squared morphology, combined with asymmetry of the neurocranium.

It has been pointed out that the uncovered crania from the Hirota Yayoi site on Tanegashima, Kagoshima Prefecture, which is the subject of this study, possess a considerable tendency for brachycephaly, as well as a sort of “tabular erect type” of modification, adopting the term defined by studies of ICM in Mesoamerica and Peru [12]. Crania from the Hirota site display lambdoidal flattening and a squared occipital deformation in the posterior part of the vault (Figs 1 and 2), as if they had been deformed by a flat device such as a board or by banding/binding, similar to that of previously mentioned international studies. Previous studies going back decades have claimed that the Hirota people may have modified their crania intentionally [20–22]. The unusual morphology of head shapes from the Hirota site crania has not been found in any other region in the Japanese archipelago [21, 22]. Furthermore, the purposes and intentions of this practice by the Hirota people have not been elucidated in any previous studies of the site.

Despite the visually distinguishable morphology of Hirota crania, to our knowledge, no studies have objectively confirmed or identified whether this unique phenomenon is in fact the result of the practice of ICM. Hirota site crania show slightly ambiguous deformed morphologies, so whether they had practiced ICM cannot be determined clearly. On the other hand, distinguishable cranial morphologies (e.g., tabular oblique, extreme tabular erect) observed in Old China, Peru, and Mexico can be conclusively understood as cases of ICM. The morphology of Hirota crania is less visually extreme in shape, which leads to the question of cultural intent versus consequence, potentially exemplified through the use of tumplines, cradleboard, and positional plagiocephaly (lopsided head) by sleeping with the back of the head facing downward. It is well known that unintentionally deformed crania can be seen in some native North American groups; such as resting a child’s head on a cradleboard [23–25].

Intentional deformation involves plastic changes in cranial shape by restricting craniofacial growth and development in certain directions, and takes place while the infant’s skull is still soft [12, 26]. As such, the aforementioned cases of ICM in North America, Mesoamerica, and South America have left “hard evidence” for those crania; therefore, ICM can be distinguishable from unintentional cranial modification, which may be the result of intensive habitual practice. Such evidence of ICM includes changes to bone structure, such as resorptive processes, porotic remodeling, and suprainiac thinning. Furthermore, many researchers have reported suprainiac depressions, circulatory bone necrosis, and infectious decays are associated with head modeling and refining among crania from the New World [27–30]. Furthermore, Tiesler and others [12, 31] have observed sagittal grooves/depressions, suprainiac depressions, and craniosynostosis (premature fusion of sutures resulting in crania asymmetry) as side effects of ICM. These multiple lines of evidence left on the crania of individuals who underwent ICM are useful frameworks or strategies when approaching the question of whether cranial deformation is indeed purposeful.

The purpose of this study is to investigate whether the lambdoidal flattening and squared/flatness of the posterior region of the crania from the Hirota site was done purposefully or resulted from unintentional habits. This study highlights the utility of three-dimensional (3D) surface scan imaging, outline-based two-dimensional (2D) geometric morphometrics (GMM), and visual assessments of cranial surface changes. To achieve this purpose, we examined abnormalities in cranial sutures that might have formed during growth and development by utilizing a novel 3D visualization method of cranial surfaces through the use of 3D mesh/cloud models.

## Materials and methods

### The Hirota archaeological site

**Fig 3:**
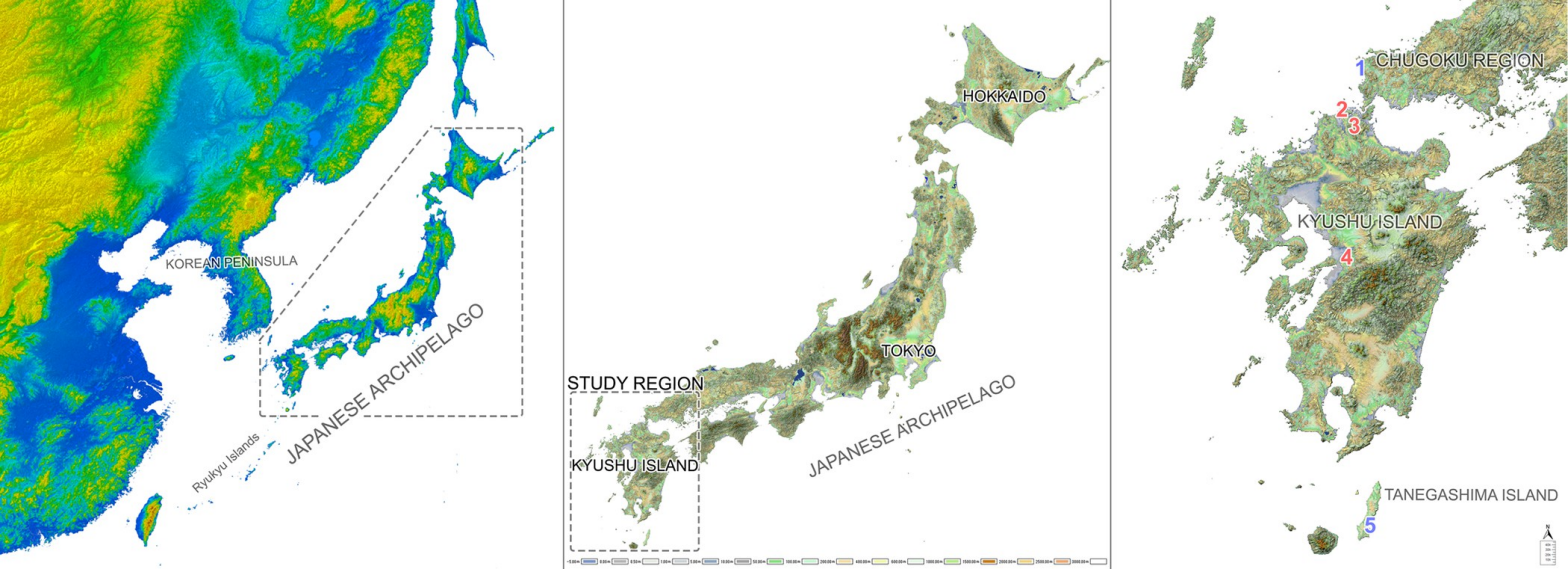
Map of the Japanese archipelago and Kyushu Island study region, including Tanagashima Island and the Yamaguchi region. Sites utilized in this study displayed as numbers: 1) Doigahama, Yayoi; 2) Yamaga, Jomon; 3) Einomaru, Jomon; 4) Goryo, Jomon; 5) Hirota, Yayoi-Kofun. Map created utilizing the ‘KASHMIR 3D’ program, utilizing public domain, open access base data from the Geospatial Information Authority of Japan (GSI).

**Fig 4:**
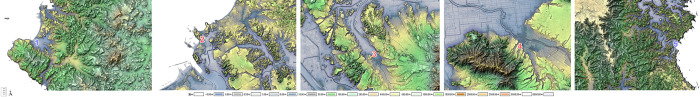
Topographic maps of each site utilized in this study and surrounding areas. Map created utilizing the ‘KASHMIR 3D’ program, utilizing public domain, open access base data from the Geospatial Information Authority of Japan (GSI).

**Fig 5:**
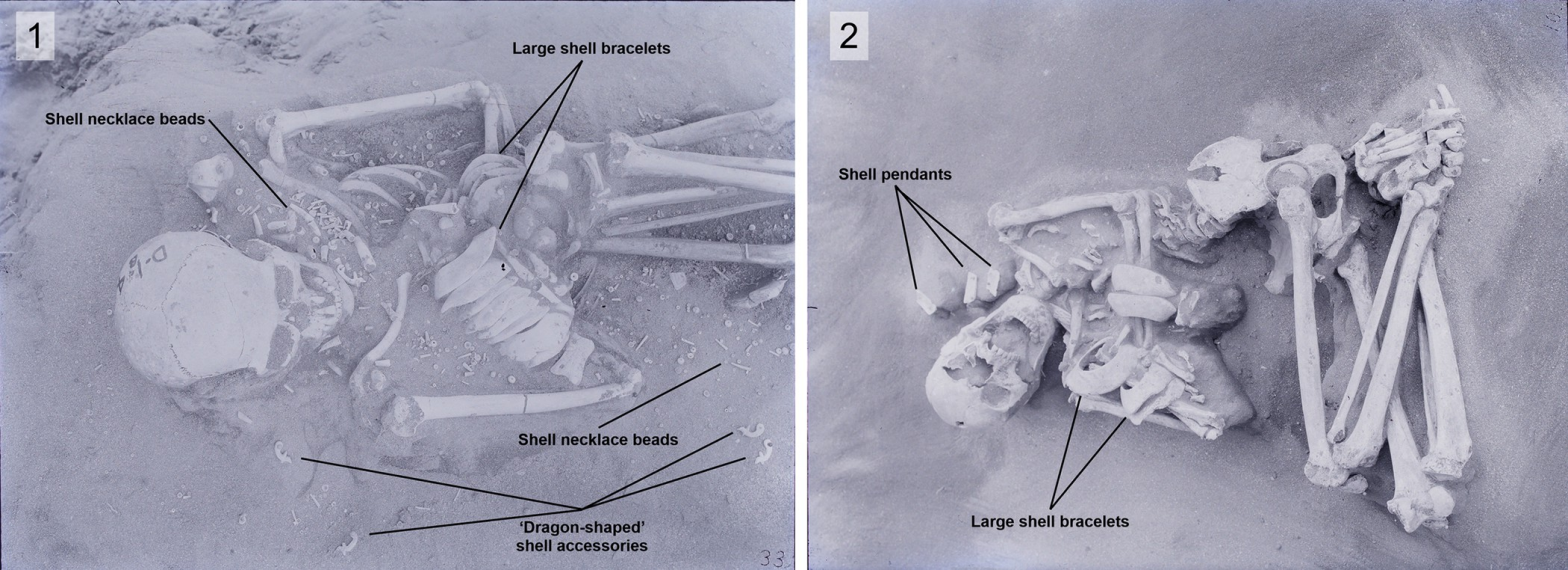
Examples of typical burials at the Hirota site, displaying large numbers of shell accessories. 1) Individual ‘HT3’ in this study; 2) Individual ‘HT9’ in this study.

The Hirota archaeological site is located on Tanegashima Island (30°25′29.5″ N, 130°58′2.2″E), one of the nine Osumi Islands belonging to Kagoshima Prefecture, Japan (Figs 3 and 4). The Hirota site is a large-scale burial site, excavated from 1957 to 1959 and again from 2005 to 2006, consisting of dozens of human burials along a coastal sand dune, where hundreds of well-preserved full and partial skeletons were uncovered along with tens of thousands of shell and glass accessories (Fig 5).

The Hirota burial ground primarily dates from the final “Yayoi” period (approximately the third century CE to the “Kofun” period around the fifth to the seventh century CE). In contrast to the immigrant-type Yayoi people from the Northern Kyushu region, who exhibit facial flatness; the craniofacial traits of the Hirota people are similar to those of “indigenous Jomon” and Ainu people, who display a prominent glabella and a strong elevated nasal ridge. However, some of their craniofacial traits differ from those of the Jomon [21]. They are characterized as short-statured as compared with the immigrant-type Yayoi people, who were tall-statured. The statures of the Hirota people were estimated as 154.0 cm on average in males and 142.8 cm on average in females [32], whereas late-period Yayoi people in Northern Kyushu exhibit statures of 161.7 cm on average in males and 157.0 cm on average in females based on the long bones [33]. Although both the Hirota and immigrant-type Yayoi people from the Northern Kyushu region practiced tooth extraction, they exhibit different extraction patterns on one side of the lateral incisor [21, 22]. Yonemoto et al. [22] investigated burial sites in the lower strata of the Hirota site and reported the presence of Wormian bones in 32 (67%) of 48 Hirota crania and porotic lesions on the parietal and occipital bones of 42%. The present study captured those porotic lesions on the cranial areas through the use of a 3D surface blue-light based scanner.

The Hirota archaeological site is particularly spectacular during this period because many shell pendants, shell bracelets, shell beads, and small glass beads were recovered, with 90% of the burials at the Hirota site containing such funeral goods [22]. In the 2005–2006 archaeological investigation, as many as 44,242 shell accessories (as seen in Fig 5) and 28 small glass beads were recovered. The shell accessories were made from green turban shells (*Turbo marmoranus*), Gohoura shells (*Strombus latissimus*), heavy frog conches, cone shells, patella shells, trumpet shells, and dentalium shells. Several types of these shells could not be found in the vicinity of the Tanegashima Island area; therefore, it was speculated that the Hirota people had been trading these shells from the south of Amami Oshima Island and even further south of the Ryukyu Islands [32, 34–37]. Furthermore, the engraved patterns on the shell pendants found in the Hirota graves are strikingly similar in style to patterns found on the mainland Chinese continent [32, 35, 36, 38, 39]. Such evidence of trade or other connections beyond Tanegashima Island, both to the south and west, suggest a high probability that the Hirota people had complex trading networks that were centered around these shell or shell artifacts.

### Materials

All human crania from the Hirota site in this study were mainly uncovered from the lower strata and are housed at Kyushu University Museum in Fukuoka, Japan. To investigate Hirota crania, we added Jomon and Doigahama Yayoi crania as comparative data. We chose Jomon crania primarily dated to the Late Jomon Period (3000–500 BCE) that were excavated from the Einomaru (33°84′33″ N, 130°73′18″E), Goryo (32° 41′ 55″N, 130°43′25″E), and Yamaga sites (33°54′35.78″N, 130°40′1.2″E) on Kyushu Island, Japan. Other comparative data are from the Doigahama Yayoi archaeological site (34°17′36.7″N, 130°53′10.4″E), which is located in Shimonoseki city, Yamaguchi, Japan. The sample size is shown in Table 1.

**Table 1:** Materials list.

Archaeological site	Cultural period	Sample size		
		Female	Male	Total
Hirota	Final Yayoi ~ Early Kofun period (third to seventh century CE)	7	12	19
Doigahama	Early to Middle Yayoi period (third century BCE-to first century CE)	11	17	28
Jomon (Einomaru, Goryo, Yamaga)	Late Jomon period (3000–500 BCE)	3	6	9
Total		21	35	56

All samples were housed in Kyushu University Museum at the time of writing. S1 Table for list of samples and original IDs from Kyushu University Museum.

### Methods

#### Ethics statement

Due to laws regulating archaeological human remains in Japan, institutional approval is granted via oral confirmation, not requiring (Institutional Review Board) IRB approval. Oral permissions have been granted in this case. Furthermore, the institute currently housing the remains utilized in this study (The Kyushu University Museum) has granted permission for data, photographic and 3D images to be displayed in this project. Human remains utilized in this study have been verified by local authorities as not having verifiable living descendants.

#### Sex and age estimation

Sex estimation of individuals is primarily based on an examination of subpubic regions, os coxae, and cranial features following the identification standards laid out by Buikstra and Ubelaker [40]. After estimating sex followed by Buikstra and Ubelaker, the results of sex estimation [21] using methods by Nakahashi and Nagai [41] were compared, confirming that our results were the same as those obtained [21] using Nakahashi and Nagai’s sex-estimation method. As these results seem to support each other, this sex-estimation method was applied in this case. The estimated age at death was based on the pubic symphysis [42] and the auricular surface of the ilium [43]. The subjects were classified into three age groups: young adult (age 20–40 years), middle adult (40–60 years), and old adult (60 years and older). Unfortunately, we could not use infants and juveniles because of the poor preservation of human remains in these groups.

#### 3D data acquisition

The cranial data utilized in this study, including 3D mesh and cloud data, were collected using a blue-light-based 3D scanner (Artec Space Spider; Artec 3D, Luxembourg city, Luxembourg). This device captures 3D data at a resolution of up to 0.1 mm and a point accuracy of up to 0.05 mm using blue-light-based technology. All scans were post-processed through a combination of Artec 15 and 16 (Artec 3D), Meshlab [44], and Geomagic Design X software (3D Systems Inc., Rock Hill, SC, USA).

The raw data for each cranium was cleaned, aligned, and merged with the superior, inferior, and 360° views taken during scanning. Once merged, the scans were further processed to remove extraneous debris and correct scanning errors. Finally, 3D mesh models (PLY files) of the crania were constructed.

#### Morphometric statistical analysis

**Fig 6:**
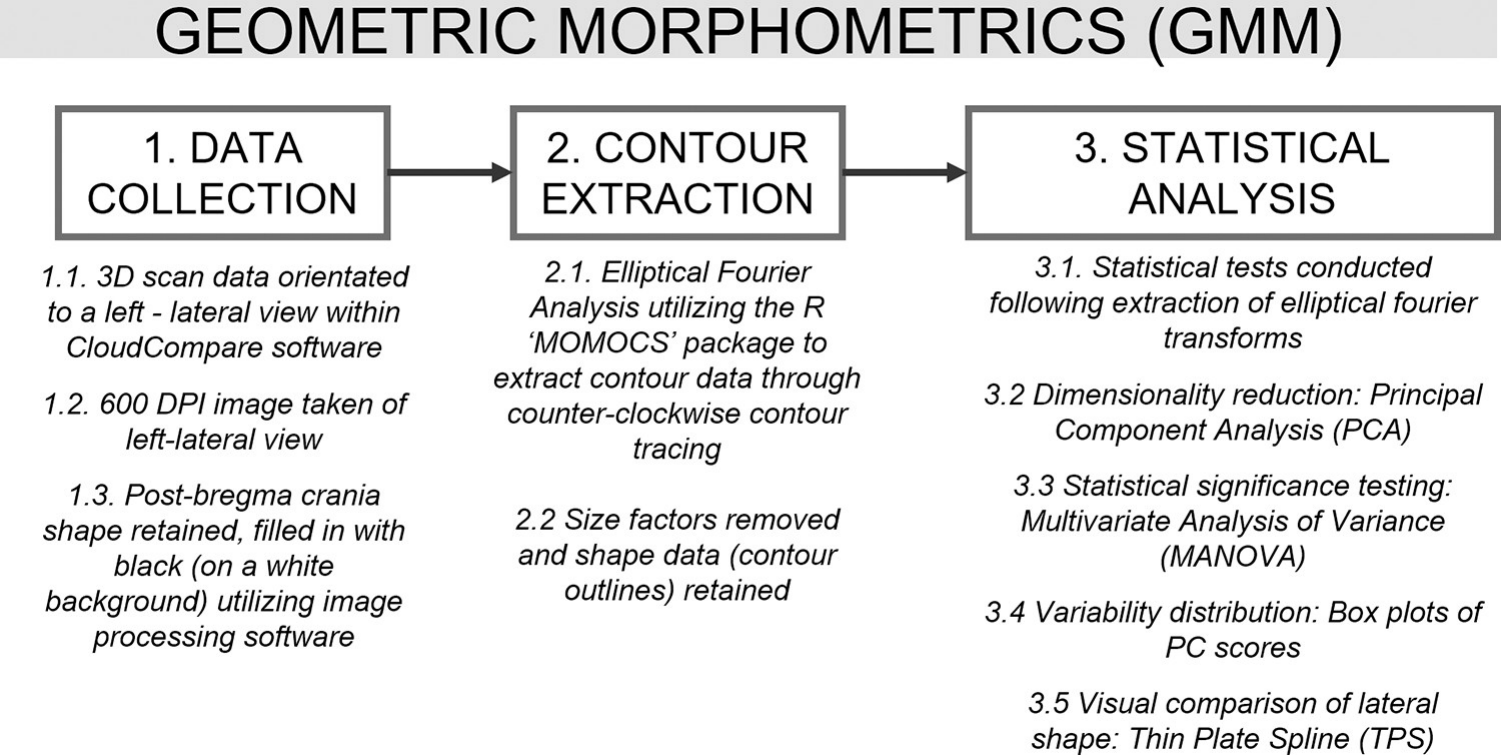
Geometric Morphometric (GMM) workflow utilized in this study.

Due to the presence of visually observable morphoscopic traits such as unusual lambdoidal flattening and occipital flatness, squared cranial shapes in posterior vaults, and a tendency toward extreme short-headedness, cranial shape outline data that could statistically illuminate such traits were extracted. These outline-based data were extracted through the following workflow (Fig 6). We oriented the 3D mesh models with the Frankfurt horizontal and mid-sagittal planes to determine the midlines. First, we extracted the sagittal plane profile from the bregma to the lambda, opisthocranion, and mastoid process area. Then, we cut off the crania perpendicular to the bregma and produced only the post-bregma outlines to the lambda and opisthocranion to opisthion. The extracted outline included the mastoid process. Next, we conducted elliptical Fourier analysis (EFA) to transform the 2D images into testable data and reveal whether the observed cranium was actually deformed/modified or within the population’s normal range of variation; we excluded the mastoid process from the obtained outline because EFA picks up the shape of the mastoid process and brow ridges. Because visually recognized extreme flatness of the occipital regions of Hirota crania is a distinctive characteristic, outline-based analysis may be a beneficial tool to quantify such whole-crania variability in shape. The posterior part of the cranium has a limited number of landmarks (parietal subtense, lambda, opisthocranion, and occipital subtense), all of which are located on sagittal outlines [45, 46]. Therefore, outline-based GMM analysis (i.e., EFA) was used in this study.

EFA aims to extract quantitative variables from 2D shapes. The ‘R’ MOMOCS package [47] (R Core Team, 2020, v3.6.3) was utilized with a code written by one of the present authors (JFL); a more detailed explanation of the utilized code and package can be found in Loftus [48]. Although 2D GMM is not a commonly utilized practice among biological anthropologists, its usefulness for quantifying the full 2D shapes of biological and archaeological objects, especially in the study of cranial morphology, is increasingly being recognized [49, 50]. Principal component analysis (PCA) and multivariate analysis of variance (MANOVA) based on the PCA scores were performed to illuminate the potential variability in cranial morphology. These allow the statistical significance between each group (sites) and sex to be quantified and tested. Thin plate splines were used to allow visual comparisons of flatness between groups (Hirota, Doigahama, and Kyushu Jomon), as well as by sex.

Visually recognizable depressions/grooves along the sagittal and lambdoidal sutures and suprainiac depression or thinning were also recorded. We utilized a novel method involving morphometric mapping (MM) and sliced segmental extraction (SSE) (see [51] for detailed workflow) to visualize these depressions on the posterior cranial surfaces. MM is a visualization tool that allows notions of thickness and curvature to be expressed through a chromatic color scale [52]. It is used most frequently in biological samples for which landmarks cannot be reliably utilized, thereby necessitating the use of a surface visualization method [51]. For example, Morimoto, Zollikofer, & Ponce de León [53, 54] utilized MM to visualize entheseal sites of muscle and ligament attachment on the external diaphyseal surfaces of the long bones in human and nonhuman species, and analyzed the topography of muscular attachment sites between taxon-specific locomotor adaptations. SSE is a novel method developed for use in this study that further extracts extremely minute variability in the cranial surface by “slicing” 3D data into horizontal bands and extracting MM on each of these bands individually, the detailed process of which is outlined further in Loftus and Seguchi [51]. MM from point clouds derived from STL mesh files of the outer surfaces of the crania were extracted using “unrolling” methods via the Cloud Compare program [55], which flattens 3D objects into a semi-2D/3D state and allows for visualization of subtle variability on the surface. Alignment points were set at the closest edge of the opening of the external auditory meatus on the mandibular fossa and opisthion. SSE was then utilized to visualize the depression/grooving along the posterior sagittal and lambdoidal sutures and suprainiac depression. These depressions/grooves are represented in a graduated color scale, with areas of depression shown in red/yellow hues, and areas of protrusion in white/blue hues for a clear visual representation of complex cranial curvatures.

## Results

### PCA and MANOVA via EFA

**Fig 7:**
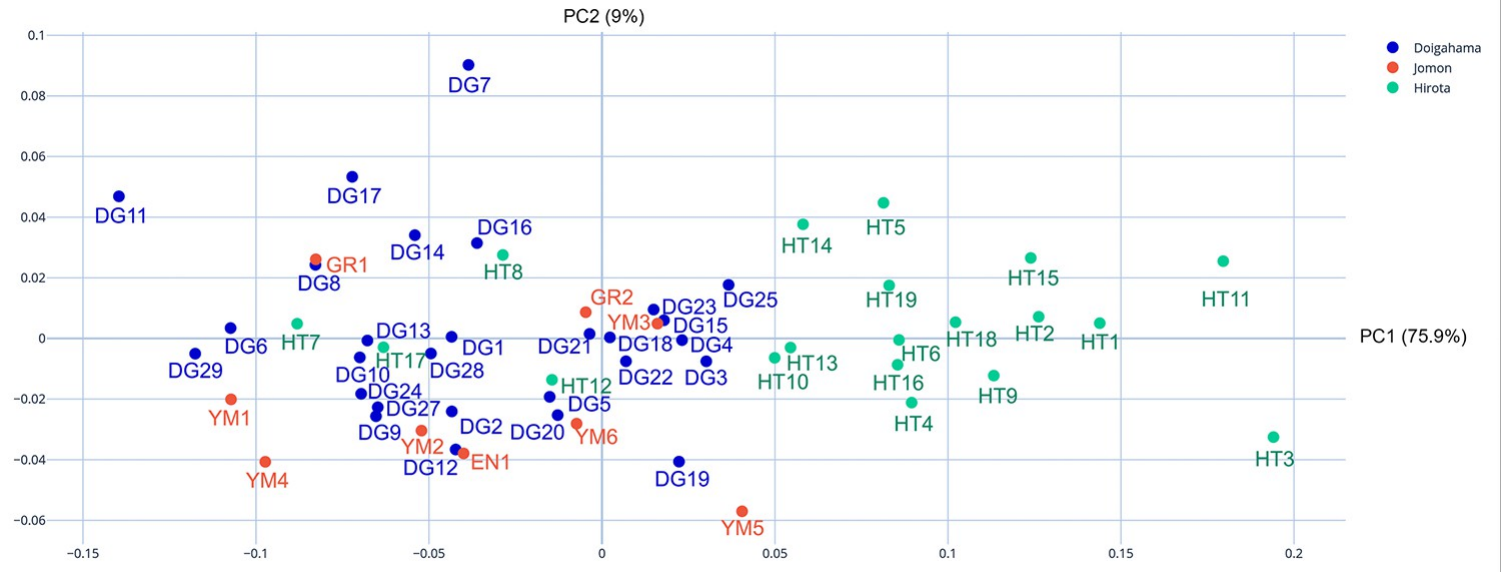
Principal component morphospace plot of the first principal component (PC1) (x axis) and PC2 (y axis). Colors denote site groupings: green: Hirota; blue: Doigahama; red: Jomon of Kyushu. PC1 and PC2 contribute 75.9% and 9.0% of the variance, respectively.

The PCA morphospace plot clearly exhibits the similarities and differences in the occipital outlines of crania by group and sex differences. The results of the PCA show clear alignment with our hypothesis that Hirota samples are morphologically different from Doigahama Yayoi and Kyushu Jomon samples. Shape variations along the principal component (PC) axes for Hirota, Doigahama, and Jomon are displayed in Fig 7 and S3 Fig. Fig 7 and S3 Fig show that there is a clear grouping in which Hirota is significantly different from Doigahama and Jomon, mostly in the right half of the morphospace, with Doigahama and Jomon crania found rather closely associated to the left (See S1 Fig for PCA of all samples, overlaid with morphospace, and S2 Fig for PCA of all samples with overlaid morphology for each sample individually, utilizing the ‘plot_PCA’ function).

**Fig 8:**
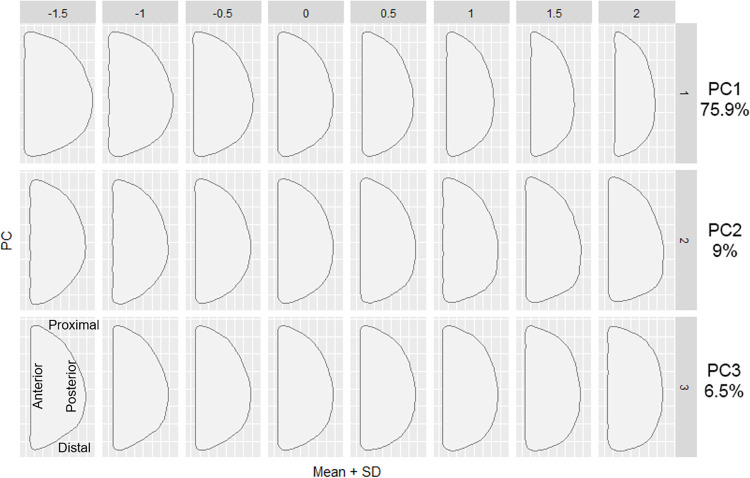
Summary of shape variation along PC axes (PC1–3) of sagittal outlines, utilizing the ‘PCcontrib’ function. The representative shape of PC1 displays flatness of the posterior parietal and occipital regions as well as the lengths from the posterior edge of the mastoid process to the posterior end of the crania. PC2 is associated with the height of the external occipital protuberance and lateral shapes of the occipital bone, such as the position and shape of the occipital bun. PC3 is associated with the flatness of the posterior cranial outlines.

The first and second PCs contributed to 84.9% of the overall variation. Fig 8 shows the shape variation along the PC axes for sagittal outlines. The first PC (PC1; eigenvector 75.9%) is associated with a flatness of the posterior parietal and occipital regions. The shape of PC1 displays flatness of the posterior parietal and occipital regions, as well as the lengths from the posterior end of the mastoid process to the posterior end of the crania. PC2 (eigenvector 9.00%) is associated with the height of the external occipital protuberance and lateral shapes of the occipital bone, such as the position and shape of the occipital bun. Doigahama and Jomon individuals heavily overlap within the PC1 and PC2 morphospaces; however, Jomon samples tend to fall more toward the negative axis in PC2 in the morphospace. PC3 (eigenvector 6.5%) is associated with the protrusion and flatness of the protuberance area of the occipital outlines. The plots of PC1, PC2, and PC3 show that Jomon samples are plotted on the negative axis of the PC3 morphospace (S3 Fig). The contribution of PC3 is very small, but Hirota samples are plotted toward a positive space exhibiting flat-headedness, but Doigahama samples are plotted toward positive and negative spaces evenly. The protrusion of the occipital protuberance area may be a characteristic of Jomon crania, although the sample numbers are small and cannot be conclusively understood as a pattern of the period in question.

In summary, there is a clear grouping of Hirota toward more “flattened” areas of the PCA morphospace, but Jomon and Doigahama are fairly similar in shape. However, Jomon show a tendency of having a so-called “occipital bun”. The left and right sides of the axis of the graph indicate longer and shorter crania, respectively. It can be understood that the majority of Hirota crania fall in this region of fairly shortened crania.

**Fig 9:**
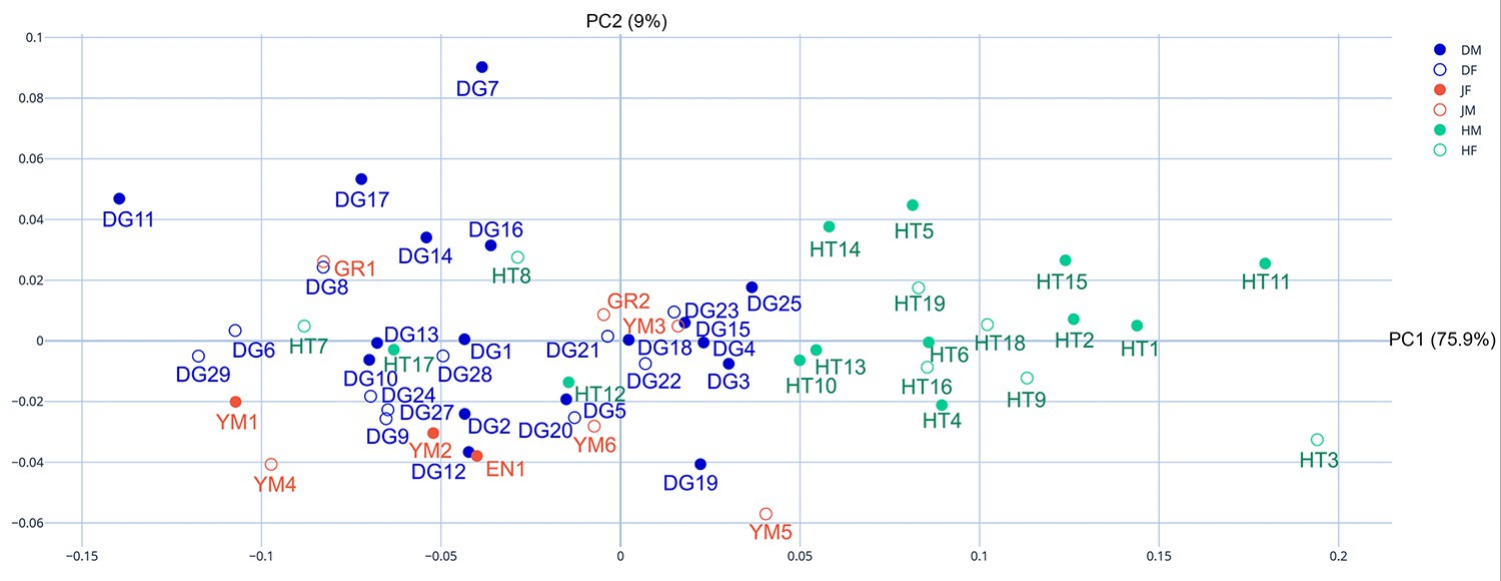
Principal component morphospace plot of PC1 (x axis) and PC2 (y axis) for sex-estimated differences between groups. Hirota: female-unfilled green, male-solid green; Doigahama: female-unfilled blue, male-solid blue; Jomon: female-unfilled red, male-solid red.

With regard to differences in sex within sites on the morphospace, Fig 9 shows the PC plot for females and males in each group. Overall, there is no clear trajectory or phenomenon of note to suggest that sex differences lead to differences in crania shape; however, Doigahama females tend to be plotted in the morphospace below zero of PC2. The results of the MANOVA utilizing the aforementioned PCA scores of 2D sagittal outlines of the Jomon–Doigahama–Hirota groups were statistically significant (F > 0.000) (Table 2). Regarding the complexity of potential individual pairwise comparisons, MANOVA overall and pairwise comparisons of sites were statistically significant (Table 3), but sex differences did not seem to be a major contributing factor to the differences in crania morphology.

**Table 2:** MANOVA output for site- and sex-based group comparisons.

COMPARISON	PILLAI’S TRACE	APPROX. F VALUE	NUM DF	DEN DF	F VALUE
SITE*	1.032	6.266	16	94	**>0.000**
SEX (Total) **	0.063	1.788	2	53	0.177

**Table 3:** Results of MANOVA pairwise comparisons for site- and sex-based group comparisons.

COMPARISON	COMPARISON	PILLAI’S TRACE	APPROX. F VALUE	NUM DF	DEN DF	F VALUE
*SITE	**DG–JM**	0.580	4.835	8	28	**>0.000**
	**DG–HT**	0.543	5.644	8	38	**>0.000**
	**JM–HT**	0.886	18.512	8	19	**>0.000**
**SEX (within sites)	DG (F)–DG (M)	0.174	2.635	2	25	0.091
	JM (F)–JM (M)	0.282	1.182	2	6	0.369
	HT (F)–HT (M)	0.043	0.365	2	16	0.699
**SEX (between sites)	**DG (F)–HT (F)**	0.536	8.687	2	15	**0.003**
	**DG (F)–HT (M)**	0.578	13.721	2	20	**0.000**
	DG (F)–JM (F)	0.355	3.036	2	11	0.089
	DG (F)–JM (M)	0.145	1.195	2	14	0.331
	DG (M)–HT (F)	0.341	5.450	2	21	0.012
	**DG (M)–HT (M)**	0.476	11.810	2	26	**0.000**
	DG (M)–JM (F)	0.391	5.471	2	17	0.014
	DG (M)–JM (M)	0.169	2.043	2	20	0.155
	**HT (F)–JM (F)**	0.913	36.738	2	7	**0.000**
	HT (F)–JM (M)	0.532	5.700	2	10	0.022
	**HT (M)–JM (F)**	0.660	11.649	2	12	**0.001**
	**HT (M)–JM (M)**	0.496	7.386	2	15	**0.005**

‘M’ = Male, ‘F’ = Female; DG = Doigahama, JM = Jomon, HT = Hirota

**only comparisons of sex between a Hirota and non-Hirota site show significance

**Fig 10:**
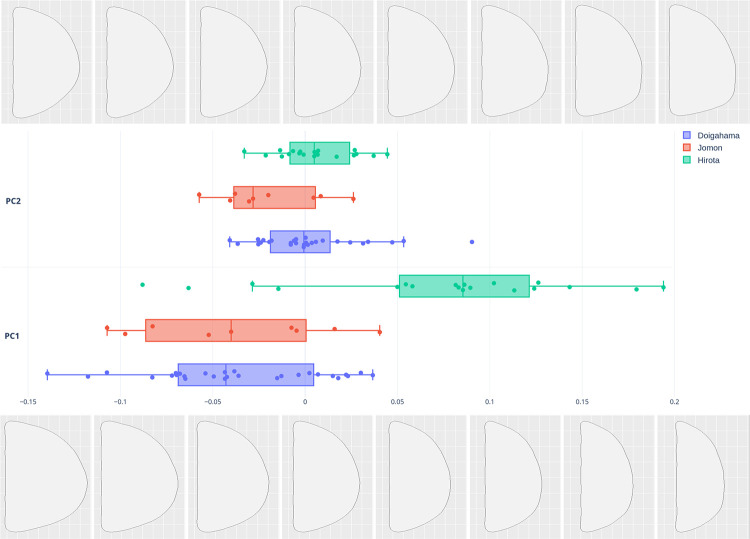
Box plots of PCA scores (PC1 (lower) and PC2 (upper)) between sites (Hirota–Jomon–Doigahama). Colors denote site groupings: green: Hirota; blue: Doigahama; red: Jomon of Kyushu. PC1 (75.9% of the variance) shows that the spread of variability and mean shape of Hirota crania are plotted differently than those of Jomon and Doigahama.

The results of MANOVA, both overall and pairwise comparisons, complemented the PC plots and illuminated not only the differences in morphology between sites, but also the less impactful role of sex in morphology. The pairwise results in particular showed that there were no statistically significant sex differences within groups, as only comparisons of sex between the Hirota and non-Hirota groups showed significant differences (Table 3). This indicates that both females and males in the Hirota group practiced ICM rather evenly. These results further support the notion that the Hirota site in particular is a morphological anomaly among the sites utilized in this study. Finally, box plots of PCA scores (PC1) showed that the Hirota scores were plotted much differently than the others (Fig 10).

**Fig 11:**
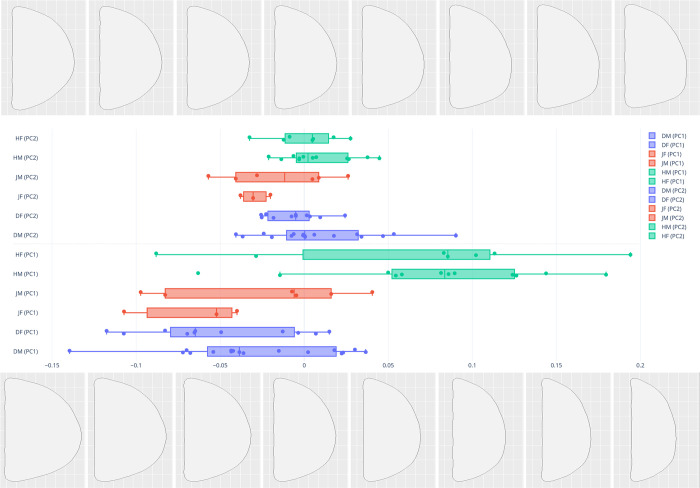
Box plots of PCA scores (PC1 (lower) and PC2 (upper)) between sex groupings (by site and sex). Colors denote site groupings: green: Hirota; blue: Doigahama; red: Jomon of Kyushu. PC1 (75.9% of the variance) shows that the spread of variability and mean shape of Hirota crania are plotted differently than those of Jomon and Doigahama.

The mean PC1 scores for Hirota were far from Jomon and Doigahama within the morphospace, but those of Jomon and Doigahama were rather similar, indicating that Hirota have shorter and flatter occipital shapes, but Jomon and Doigahama display longer and less flat shapes. Box plots of PC2 scores show overlapping among Hirota, Jomon, and Doigahama; however, the mean of Jomon is different from that of Hirota and Doigahama. The PC2 scores for Jomon show a characteristic in which the occipital bone is more protruded compared with Doigahama and Hirota. Fig 11 shows box plots of PCA scores for sex differences in each group, with both males and females of the Hirota site showing clear uniqueness toward a flattened occipital region, further visualizing the evenness of potential ICM between sexes at the site.

### Thin plate splines

**Fig 12:**
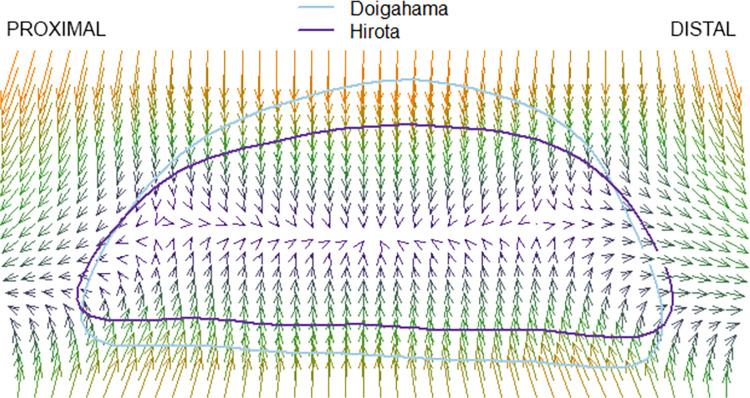
Comparative thin plate spline output (Hirota and Doigahama). Hirota shows shorter and flattened occipital outlines.

**Fig 13:**
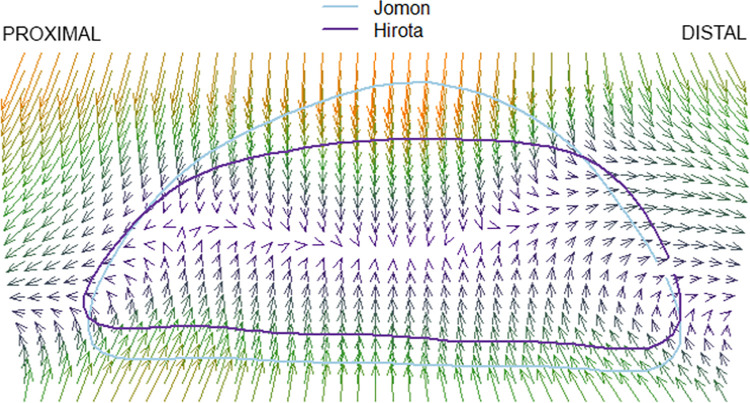
Comparative thin plate spline output (Hirota and Jomon). Hirota shows shorter and flattened occipital outlines.

**Fig 14:**
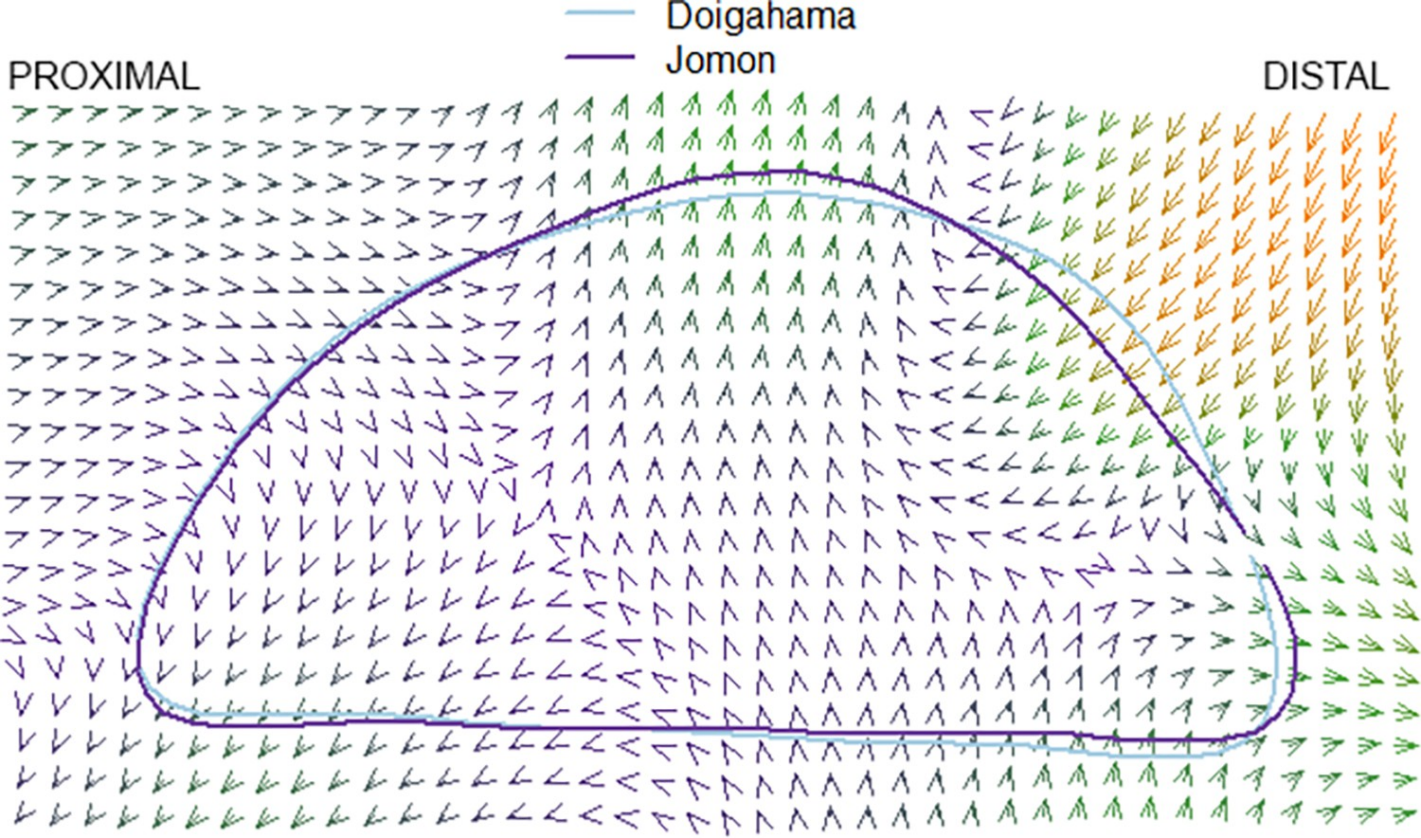
Comparative thin plate spline output (Hirota and Doigahama). Doigahama and Jomon show similar occipital shapes.

**Fig 15:**
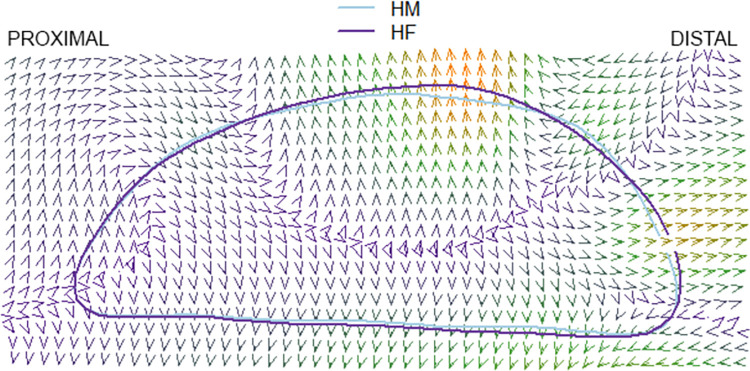
Comparative thin plate spline output (male and female Hirota).

**Fig 16:**
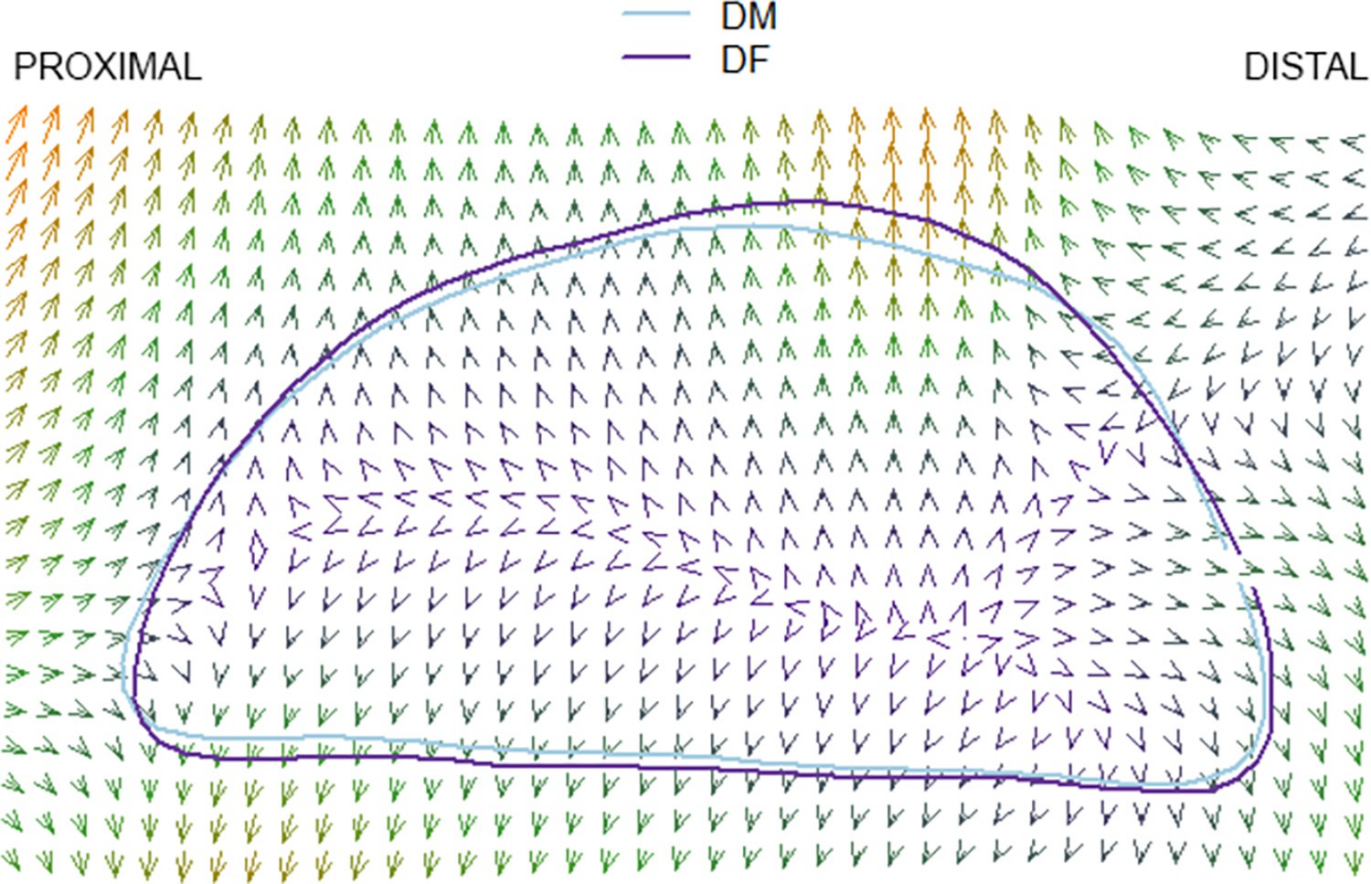
Comparative thin plate spline output (male and female Doigahama).

**Fig 17:**
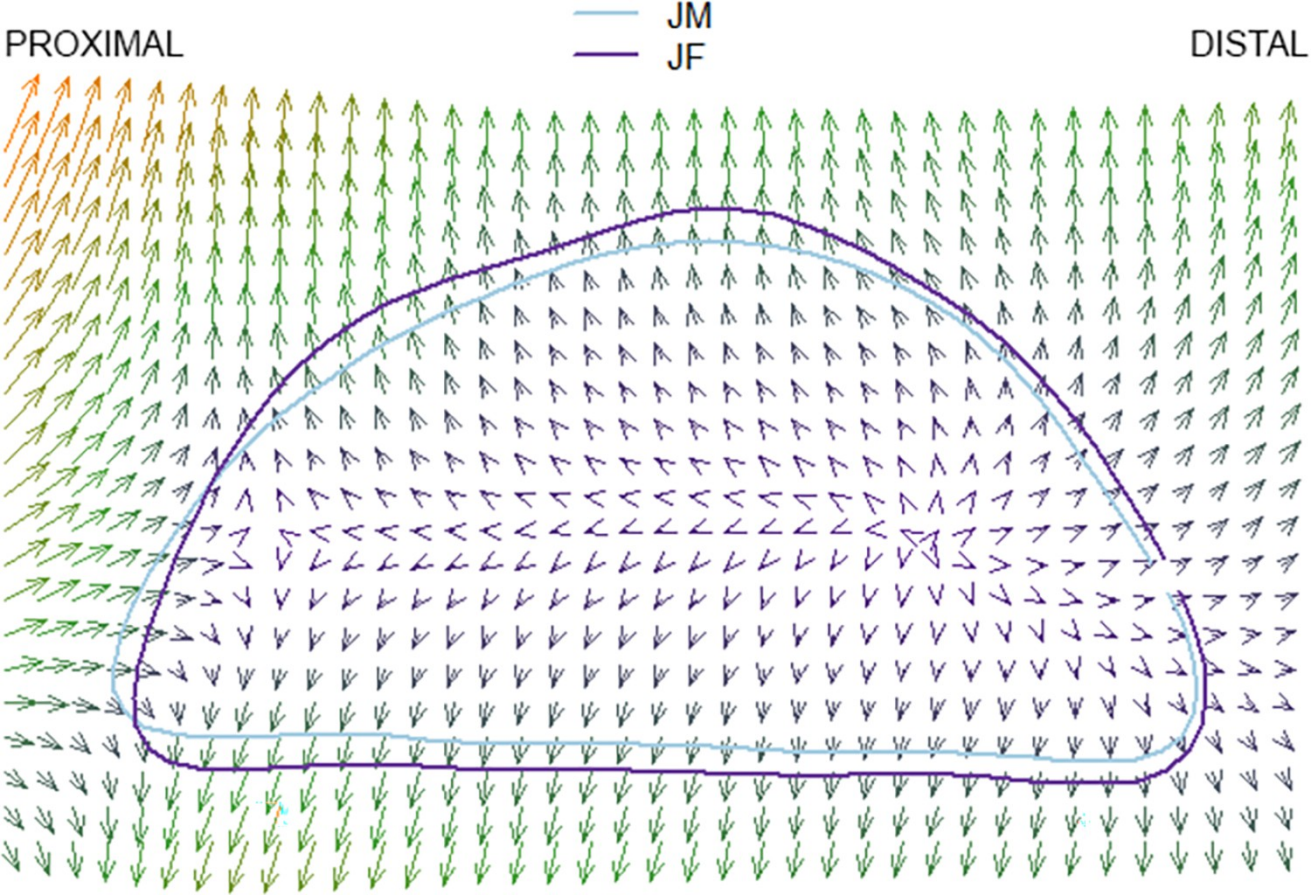
Comparative thin plate spline output (male and female Jomon).

The results of the pairwise comparison of thin plate splines are as follows: Hirota vs. Doigahama, Hirota vs. Jomon, and Jomon vs. Doigahama are shown in Figs 12–17. These outlines visually indicate that the shape of Hirota is different from those of Doigahama and Jomon, with the shapes of Jomon and Doigahama being similar and mirroring the morphological results of 2D GMM. In general, the occipital shapes of Hirota are flatter than those of Doigahama and Jomon. Moreover, Doigahama individuals have protruded areas in the occipital bone more distally than do Jomon individuals. Figs 15–17 show the thin plate spline of sex differences in each group. No distinct sex difference in occipital shapes was found within each group. Both females and males of the Hirota samples show flat and short occipital shapes, also mirroring the 2D GMM results, which show no clear variability between males and females in this area of the crania.

### Visualizing sagittal and lambda depressions/grooves using MM and SSE

ICM can be distinguished from UCM, in which crania might be deformed by intensive habitual practice, using “hard evidence” (previously outlined in section 1 ‘Introduction’) such as sagittal grooves or a strong sagittal sulcus dividing the parietals on crania, as well as suprainiac thinning or depressions on the occipital bone [12]. This study also recognized depressions along the posterior portion of the sagittal and lambdoidal sutures, as well as suprainiac depressions on Hirota site crania. On the other hand, sagittal/lambdoidal and suprainiac depressions were not observed on Doigahama and Jomon crania.

**Fig 18:**
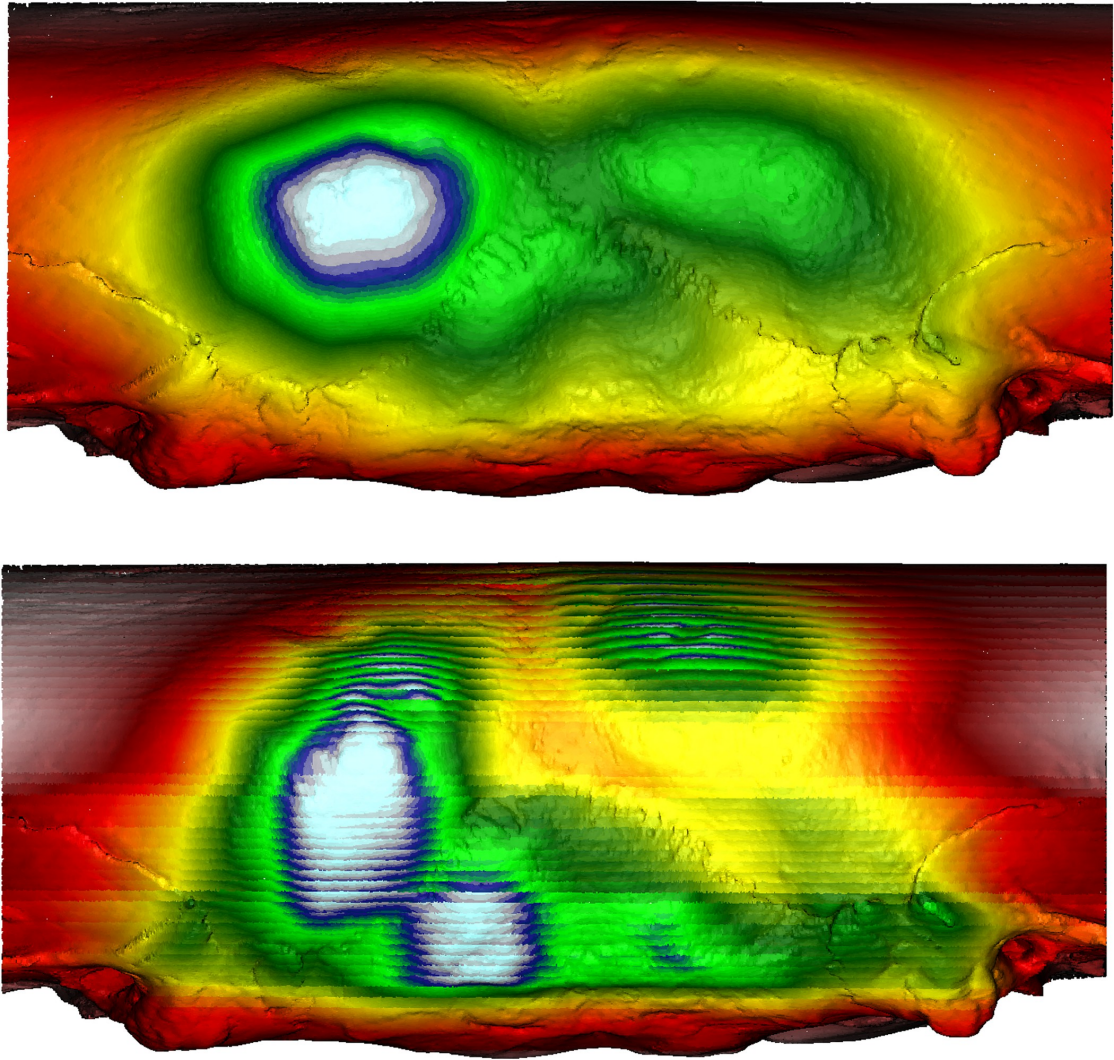
Morphometric mapping (upper) and sliced segmental extraction (lower) of sample HT 16. It has flat and short crania displaying depressions along the sagittal and lambdoidal sutures. MM captures the suprainiac depression and depressions on the right side of the lambdoidal suture. SSE captures the depression of the sagittal suture, shown in yellow/orange. The occipital area shows asymmetrical protrusion, shown in white/blue. Color representations: areas of depression red<yellow<green<blue<white areas of protrusion.

**Fig 19:**
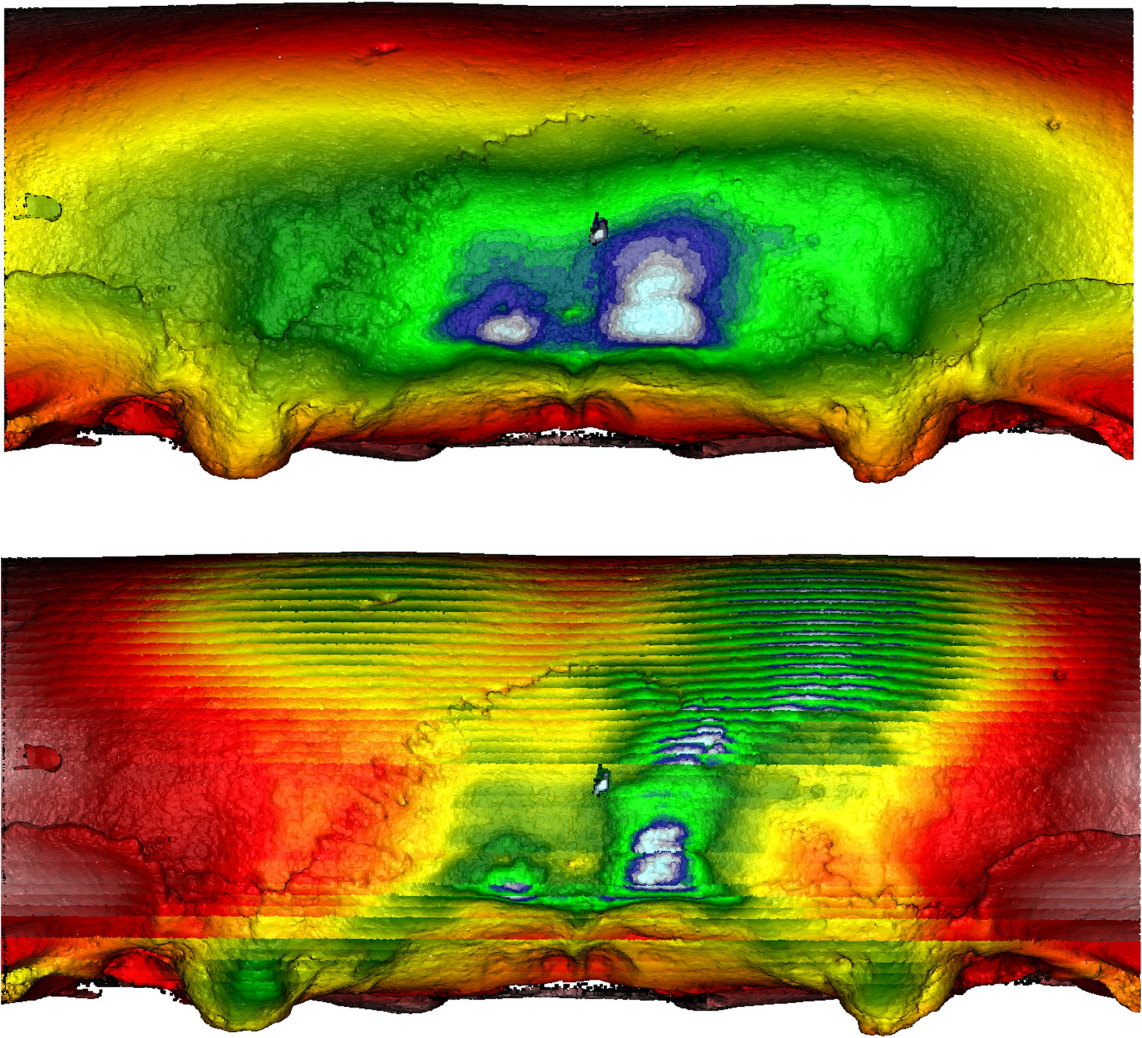
Morphometric mapping (upper) and sliced segmental extraction (lower) of sample HT 12. It has flat and short crania displaying depressions along the sagittal and lambdoidal sutures. MM and SSE capture the suprainiac depression and depressions on the sagittal and lambdoidal sutures. Both sides of the suprainiac depression show two projections, shown in yellow/orange. The occipital area shows asymmetrical protrusion, shown in white/blue. Color representations: areas of depression red<yellow<green<blue<white areas of protrusion.

**Fig 20:**
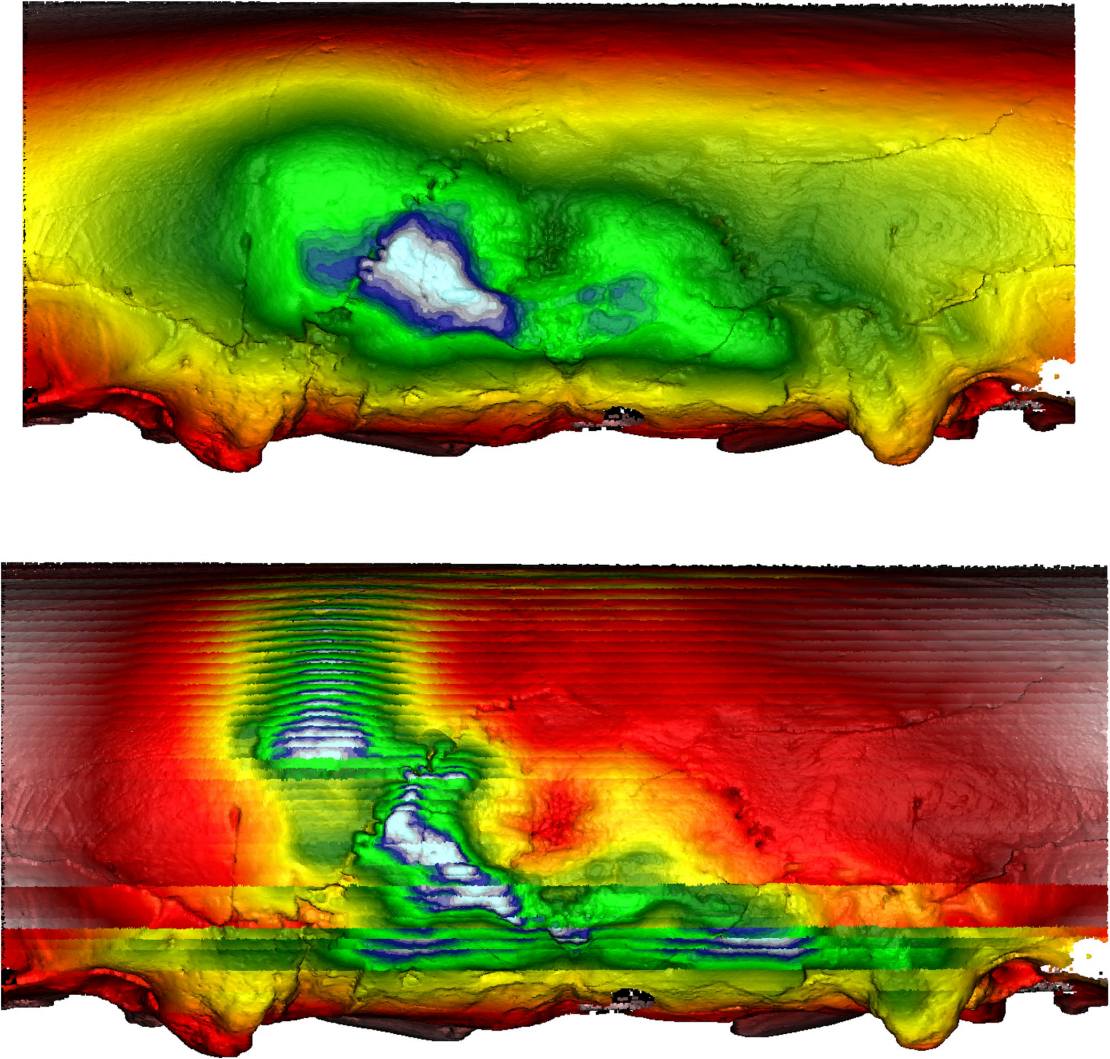
Morphometric mapping (upper) and sliced segmental extraction (lower) of sample HT 6. It has flat and short crania displaying depressions along the sagittal and lambdoidal sutures. MM and SSE capture the suprainiac depression and depressions on the sagittal and lambdoidal sutures, shown in yellow/orange. Both sides of the suprainiac depression show two “mountain-like” protrusions. The occipital area shows asymmetrical protrusion, shown in white/blue. Color representations: areas of depression red<yellow<green<blue<white areas of protrusion.

**Fig 21:**
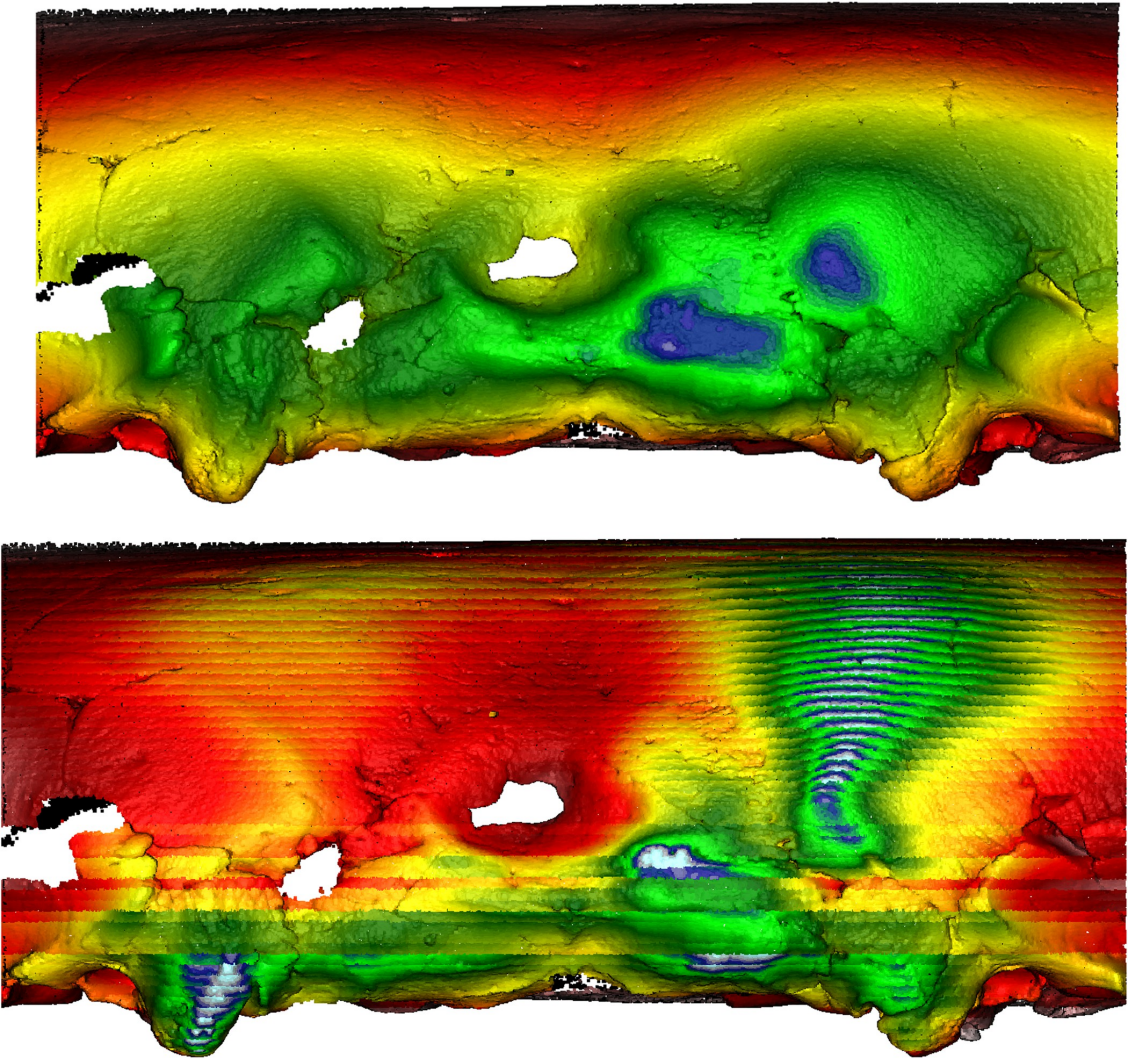
Morphometric mapping (upper) and sliced segmental extraction (lower) of sample HT 15. It has a flat, short crania displaying suprainiac thinning (as demonstrated by the hole and surrounding area of visible thinning showing concaved depression). Depressions along the sagittal and lambdoidal sutures are also present. MM and SSE capture suprainiac thinning and depressions on the sagittal and lambdoidal sutures, shown in yellow/orange. The occipital area shows asymmetrical protrusion, shown in white/blue. Color representations: areas of depression red<yellow<green<blue<white areas of protrusion.

We utilized our novel MM and SSE method [51] to visualize the “hard evidence” of potential ICM on the posterior surfaces of crania. The gradient colors of the MM/SSE outputs are direct correlations with the degree of inward/outward curvature, with red/yellow hues representative of areas of depression and white/blue hues representative of outward protrusions. The following passage outlines the best examples of such evidence within Hirota samples as compared with seemingly undeformed Doigahama and Jomon samples. The cranium of one individual (Hirota 16) had a depression along the posterior portion of the sagittal and lambdoidal sutures. MM and SSE showed those depressions along the sagittal and lambdoidal sutures clearly, represented by the “valley-like” representation of morphology shown through the color represented (Fig 18; See other examples of Figs 19–21).

**Fig 22:**
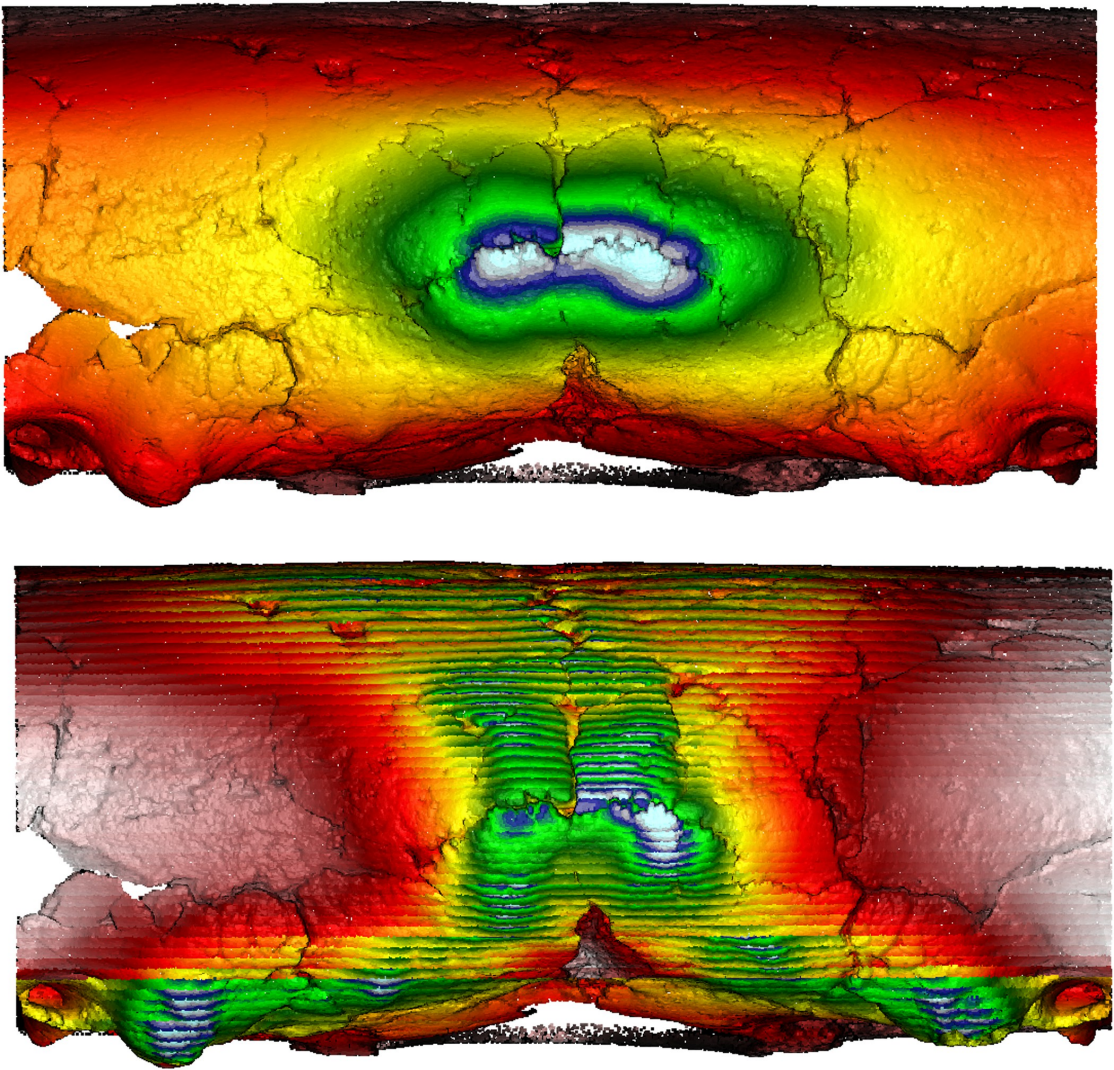
Morphometric mapping (upper) and sliced segmental extraction (lower) of sample DG29. It shows no evidence of intentional cranial modification, as displayed by the lack of depressions along the sagittal and lambdoidal sutures. Color representations: areas of depression red<yellow<green<blue<white areas of protrusion.

**Fig 23:**
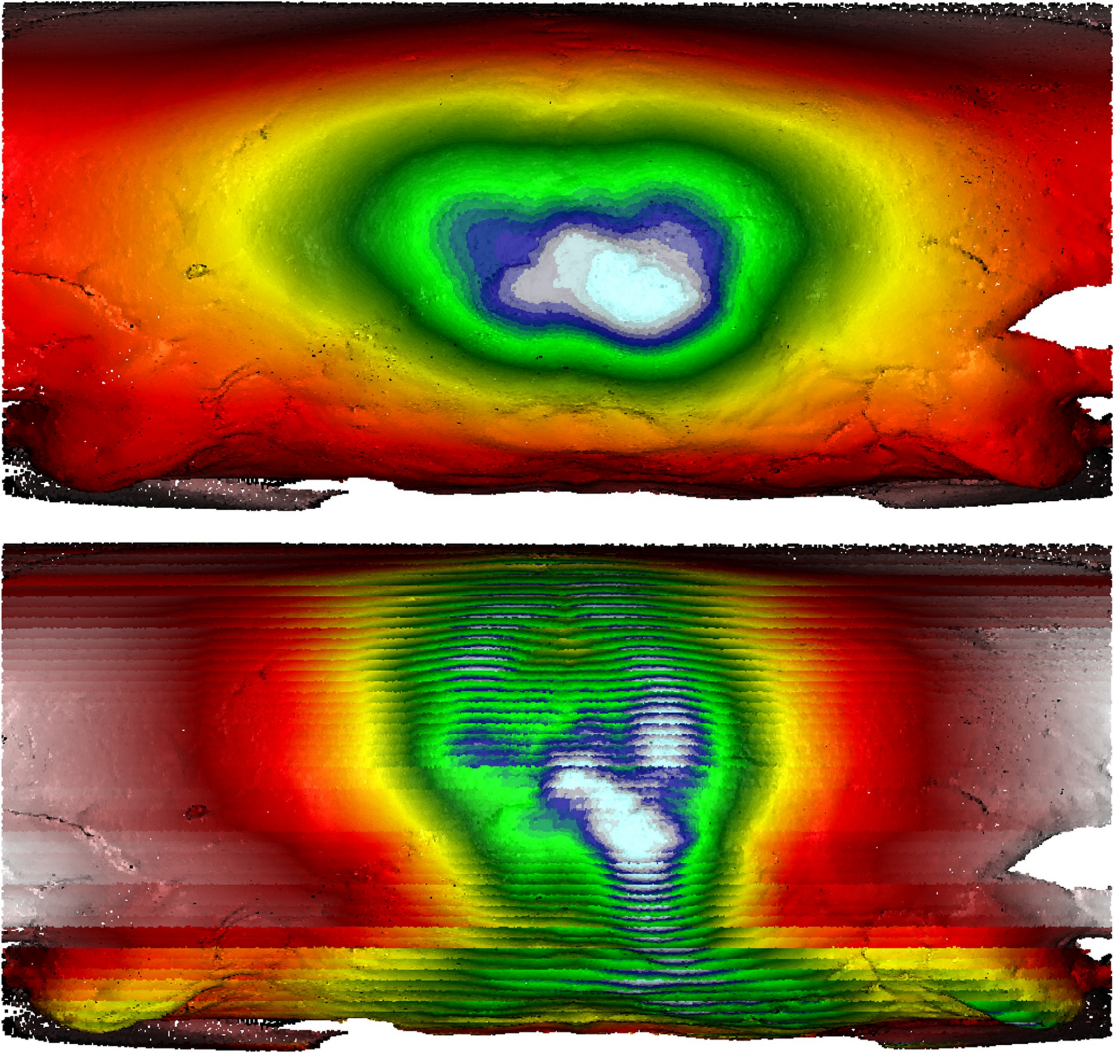
Morphometric mapping (upper) and sliced segmental extraction (lower) of sample Jomon EN1. It shows no evidence of intentional cranial modification, as displayed by the lack of depressions along sagittal and lambdoidal sutures. Color representations: areas of depression red<yellow<green<blue<white areas of protrusion.

However, as a comparison, the cranium of another individual (Doigahama 29) did not show any such depressions along the projected occipital area (Fig 22). Furthermore, the cranium of an individual from the Jomon group (Einomaru 1) also exhibited no depressions along the sagittal and lambdoidal sutures (Fig 23). Due to the tendency of depressions to be along the sagittal and lambdoidal sutures in Hirota individuals, two peak “mountain-shaped” protrusions with visibly noticeable asymmetry in the posterior region are noted as common among Hirota crania. Doigahama and Jomon individuals do not show such protrusions and asymmetry, but rather, a normal, singular, central protrusion for which the morphology gradually blends into the surrounding region. On the other hand, the protrusions on Hirota crania are often asymmetrical in regard to the amount of protrusion, the positioning of which also seems to be asymmetrical in nature, with the vertical placement of the most protruding areas being different, even in the same individual.

## Discussion

Based on the above results, a visually recognizable and statistically significant variability existed between Hirota crania samples and the compared Jomon and Doigahama Yayoi individuals. In addition, 2D GMM indicated that a large majority of the Hirota samples were defined by a flattened occipital region of the crania, and were morphologically very distinct during and between study periods. When utilizing 3D MM and SSE, the Hirota crania showed unusual depressions along the sagittal and lambdoidal sutures, and some showed obvious suprainiac depressions. The extreme flatness of these head shapes combined with the international literature supporting similar patterns of observable traces in individuals who practiced ICM suggest that the Hirota people most likely practiced ICM, albeit in a subtler manner than other more flamboyant international examples.

With the question of the objective uniqueness of Hirota cranial morphology settled, and strong evidence supporting ICM at the site outlined, further questions arise about whom, why, and how the Hirota people practiced ICM, with cross-cultural biological and archaeological evidence elucidating several possibilities. Previous studies have concluded that ICM was practiced among the elites in Classic Maya in Mesoamerica (ca. AD 250–900), which had rigidly divided social structures. Nagaoka, Seki, Hidalgo, & Chocano [56] (2020) used artificially deformed crania as one of the determinant factors of the elite of Pacopampa (3000–50 BCE), which are associated with mineral pigments, precious ornaments, and goods in Peru. However, recent surveys by Tiesler and Benedict [57] deny any elite associations between cranial modification. Tiesler [12] reported that more than 80% of the settlers of the coastal and inland territories of Yucatan showed artificially modified crania.

The Yayoi period is regarded as a pre-state society, whereas the Kofun period led to a rise in social complexity and the emergence of a state society. Furthermore, the development of social stratification did not develop at the same pace within the Japanese archipelago, and may have been seen earlier or later in certain central or peripheral regions. Evidence of increases in violence, grave goods, and variability in mortuary treatment may reflect social class or social stratification [58, 59]. However, 90% of burials at the Hirota site were associated with funeral goods, such as shell ornamental products. In contrast to HT7, HT8, HT17, and HT12, which are plotted within the range of the Doigahama samples, all other Hirota crania are plotted separately from the Doigahama Yayoi and Jomon samples. HT12 has a slightly longer skull, however it still indicates intended cranial modification showing the flat occipitals as seen in 3D methods. HT17 was an interesting case that still showed cranial deformation, but whose burial did not contain burial goods and had a strontium value that differed from other Hirota samples [22]. Unfortunately, the study of strontium was performed with the individuals who retained teeth, especially with upper incisors. The strontium values were not obtained from many of the skulls we tested for intentional cranial deformation practice. The strontium values were only obtained from HT4, HT13, and HT17 [22]. The specific sex-based dominance of deformation practices was not statistically significant, indicating that both Hirota females and males practiced ICM. It is difficult to determine whether the Hirota society had clear social stratification. At the current excavation stage, it is difficult to determine whether their society was rigidly ranked because most of their burials were associated with rich funeral goods made using shellfish brought from hundreds or thousands of kilometers away. It may be possible that almost all Hirota individuals enjoyed such decorated burials and ICM evenly because of having no social strata. Alternatively, it is feasible to claim that only the graves of the elite class were found at the Hirota site.

Doigahama and other Yayoi rice agriculturalists in Northern Kyushu show more developed entheses on the lower extremities compared with Hirota individuals, and it appears that this pattern of entheses is characteristic of Yayoi agriculturalists [22, 60]. This evidence may support the idea that the Hirota people did not partake in rice agriculture on this island. Yonemoto et al. [22] suggested that the Hirota people caught fish in the nearby coral reef for sustenance. It is widely understood that social stratification in Japan was not widely adopted until the adoption of a rice cultivation society. It is only after rice cultivation became a main means of providing sustenance that cooperative work in wet rice paddy fields increased and settlements became larger and more concentrated and a communal network of people, goods, and information began to accumulate, leading to increased observable evidence of social strata [61]. According to archaeological and biological evidence supporting the notion that the Hirota population did not partake in rice agriculture, it appears that their ICM practice was not associated with social ranking or gender.

After eliminating possibilities of sex- and social strata-based deformation practices, the question remains regarding the purpose of this ICM practice among the Hirota population. As previously mentioned, beyond cranial morphology, another unique characteristic of the Hirota people is that they had extraordinary accessories made of shells. As these shells could not be collected from their region of residence, the Hirota people must have traded shell accessories and products from the south of Tanegashima Island toward the Ryukyu Islands (Kinoshita, 1996). It is possible that cranial deformations were performed to create a group identity and facilitate trade of these shell goods. Such results would not be uncommon among prehistoric cultures that practiced ICM, and due to the absence of other possible reasons for ICM practices, we hypothesize that the Hirota people did so for this purpose; however, this could change if future excavations of the region were to provide evidence of either a rice cultivation or stratified society.

In terms of what could not be fully explained by utilizing the methods of this study, the Hirota people’s intentionally modified crania were identified based on the presence of significantly flattened occipitals. Those crania are also associated with lateral bulging of the parietals near the lambdoid suture [5] and display noticeable cranial asymmetry. However, although landmark-based analysis was attempted along this line, we were unable to obtain statistically significant differences in MANOVA between the Hirota and other groups, despite the Hirota being clearly separate from the Jomon and Doigahama samples in the PC morphospace. This was likely due to a lack of captured data points using landmarks. Therefore, in future studies of cranial deformation, a more clearly defined method of capturing potential cranial asymmetry (either through a more complexified landmark-based method or otherwise) should be included as a hybridized workflow to accompany the 2D GMM and 3D MM/SSE methods utilized in the present study. Furthermore, sex estimation methods utilized in this study [40, 41] could be understood as a more traditional approach to estimating sex in prehistoric populations. While more ‘modern’ approaches do exist, the applicability of these in prehistoric Asian (especially Japanese) populations has yet to be fully understood in previous research. Furthermore, in prehistoric data sets such as the Hirota samples utilized in this study which do contain well preserved os coxae for all samples, new sex estimation methods such as DSP2 [62] are difficult to utilize at the current juncture. While the authors stand by the usage of more traditional approaches to sex estimation within the scope of this study, it may be beneficial to the wider field to highlight potential differences in sex estimation methods in future studies of prehistoric Japanese populations.

Previous reports of Hirota cranial deformation used morphoscopic observation without morphometric analysis; therefore, subjectivity was involved in the determination of ICM. The present study applied a hybridized 2D outline-based GMM analysis and visualization of 3D micro-curvature on the cranial surface. Based on a comprehensive review of all the results, we concluded that the crania found at the Hirota site were intentionally modified. Although it is not currently possible to determine conclusively that there was no social hierarchy during this period, it appears that the cranial modification practice at the Hirota site was not associated with gender or social rank. However, the motivation for the practice is still unclear because of a lack of sex- and class-based evidence. The Hirota people may have preserved their group identity by modifying their crania to aid in the shellfish trade, which ended up lavishly adorning their bodies in death.

## Limitations of this study

It is important to discuss the origins and admixture of populations of the Hirota site with the Kyushu Jomon people and Doigahama people in terms of phylogeny. The skull morphology and nonmetric dental trait of the Hirota site inhabitants show similarities to the Jomon and Ainu [21, 63], however it is still unknown whether they are related to the prehistoric Ryukyu Islands people who are believed to have traded using shellfish, the Doigahama people, or the people of the Jomon period. Paleogenomic analyses of the Hirota, Kyushu Jomon, and the Doigahama have not been conducted yet. The analysis of the mitochondrial DNA of a single Hirota remains belonging to the Kofun period excavated by the Minamitane-cho Board of Education in 2005 (which differs from the Hirota site human remains we analyzed here) was conducted. It was identified as B4f, a haplogroup that had never been found in Jomon people before [64]. Shinoda et al. [64] state that the only way to accurately reveal the origin of this individual is to analyze nuclear DNA. We must wait for further paleogenomics research on the human remains excavated from the Hirota site in the future to comprehend the trade relations of shellfish products at Hirota and the geneflow/migration of people associated with them.

## Supporting information

S1 Table List of sample and original IDs from Kyushu University Museum. (DOCX)

S2 Table Minimal Data Set which supports the findings of this study. (DOCX)

S1 Fig Principal component analysis (PCA) of all samples, overlaid with morphospace. (TIF)

S2 Fig Principal component analysis (PCA) of all samples, with overlaid morphology for each sample individually, utilizing the ‘plot_PCA’ function. (TIF)

S3 Fig Principal component morphospace plot of the first principal component (PC1) (x axis), PC2 (y axis), and PC3 (z axis). Colors denote site groupings: green: Hirota; blue: Doigahama; red: Jomon of Kyushu. PC1, PC2, and PC3 contribute 75.9%, 9.0%, and 6.5% of the variance, respectively.
(TIF)
