# Supplementary material for: Investigating intentional cranial modification: A hybridized two-dimensional/three-dimensional study of the Hirota site, Tanegashima, Japan
Source: PLoS One. 2023 Aug 16;18(8):e0289219. doi: 10.1371/journal.pone.0289219 (PMC10431670; doi:10.1371/journal.pone.0289219)
Supplement: S1 Table — (DOCX) [file pone.0289219.s001.docx]

**S1 Table**

| **ID** | **SITE** | **JOMON–DOIGAHAMA–HIROTA** | **SEX** | **ORIGINAL ID** | **SITE-SEX** |
| --- | --- | --- | --- | --- | --- |
| DG1 | Doigahama | Doigahama | Male | Doigahama1001A | DM |
| DG10 | Doigahama | Doigahama | Male | Doigahama1108A | DM |
| DG11 | Doigahama | Doigahama | Male | Doigahama1116 | DM |
| DG12 | Doigahama | Doigahama | Male | Doigahama125 | DM |
| DG13 | Doigahama | Doigahama | Male | Doigahama126 | DM |
| DG14 | Doigahama | Doigahama | Male | Doigahama130 | DM |
| DG15 | Doigahama | Doigahama | Male | Doigahama136 | DM |
| DG16 | Doigahama | Doigahama | Male | Doigahama140 | DM |
| DG17 | Doigahama | Doigahama | Male | Doigahama201B5 | DM |
| DG18 | Doigahama | Doigahama | Male | Doigahama221 | DM |
| DG19 | Doigahama | Doigahama | Male | Doigahama225 | DM |
| DG2 | Doigahama | Doigahama | Male | Doigahama2A1311 | DM |
| DG20 | Doigahama | Doigahama | Female | Doigahama244 | DF |
| DG21 | Doigahama | Doigahama | Female | Doigahama303 | DF |
| DG22 | Doigahama | Doigahama | Female | Doigahama310 | DF |
| DG23 | Doigahama | Doigahama | Female | Doigahama311 | DF |
| DG24 | Doigahama | Doigahama | Female | Doigahama313 | DF |
| DG25 | Doigahama | Doigahama | Male | Doigahama408 | DM |
| DG27 | Doigahama | Doigahama | Female | Doigahama417 | DF |
| DG28 | Doigahama | Doigahama | Female | Doigahama809 | DF |
| DG29 | Doigahama | Doigahama | Female | DoigahamaB1061112M | DF |
| DG3 | Doigahama | Doigahama | Male | Doigahama222 | DM |
| DG4 | Doigahama | Doigahama | Male | Doigahama250 | DM |
| DG5 | Doigahama | Doigahama | Male | Doigahama413 | DM |
| DG6 | Doigahama | Doigahama | Female | Doigahama422 | DF |
| DG7 | Doigahama | Doigahama | Male | Doigahama1003 | DM |
| DG8 | Doigahama | Doigahama | Female | Doigahama1004A | DF |
| DG9 | Doigahama | Doigahama | Female | Doigahama1102 | DF |
| EN1 | Einomaru | Jomon | Female | FKEinomaru6 | JF |
| GR1 | Goryo | Jomon | Male | FKGoryo1 | JM |
| GR2 | Goryo | Jomon | Male | FKGoryoII2 | JM |
| HT1 | Hirota | Hirota | Male | HirotaA8 | HM |
| HT10 | Hirota | Hirota | Male | HirotaE61 | HM |
| HT11 | Hirota | Hirota | Male | HirotaC2 | HM |
| HT12 | Hirota | Hirota | Male | HirotaC6 | HM |
| HT13 | Hirota | Hirota | Male | HirotaC8 | HM |
| HT14 | Hirota | Hirota | Male | HirotaD15 | HM |
| HT15 | Hirota | Hirota | Male | HirotaD1B5 | HM |
| HT16 | Hirota | Hirota | Female | HirotaD23 | HF |
| HT17 | Hirota | Hirota | Male | HirotaD25 | HM |
| HT18 | Hirota | Hirota | Female | HirotaE31 | HF |
| HT19 | Hirota | Hirota | Female | HirotaN2E | HF |
| HT2 | Hirota | Hirota | Male | HirotaBN2W | HM |
| HT3 | Hirota | Hirota | Female | HirotaD14 | HF |
| HT4 | Hirota | Hirota | Male | HirotaD32 | HM |
| HT5 | Hirota | Hirota | Male | HirotaD41E4 | HM |
| HT6 | Hirota | Hirota | Male | HirotaD91 | HM |
| HT7 | Hirota | Hirota | Female | HirotaE1C148 | HF |
| HT8 | Hirota | Hirota | Female | HirotaE3 | HF |
| HT9 | Hirota | Hirota | Female | HirotaE41E21 | HF |
| YM1 | Yamaga | Jomon | Female | FKYamaga16 | JF |
| YM2 | Yamaga | Jomon | Female | FKYamaga17 | JF |
| YM3 | Yamaga | Jomon | Male | FKYamaga18 | JM |
| YM4 | Yamaga | Jomon | Male | FKyamaga7 | JM |
| YM5 | Yamaga | Jomon | Male | FKYamaga9 | JM |
| YM6 | Yamaga | Jomon | Male | Yamaga15 | JM |

S1 Table. List of sample and original IDs from Kyushu University Museum
