# Supplementary material for: Investigating intentional cranial modification: A hybridized two-dimensional/three-dimensional study of the Hirota site, Tanegashima, Japan
Source: PLoS One. 2023 Aug 16;18(8):e0289219. doi: 10.1371/journal.pone.0289219 (PMC10431670; doi:10.1371/journal.pone.0289219)
Supplement: S2 Table — (DOCX) [file pone.0289219.s002.docx]

**S2 Table**

| **Fig#** | **STAT. METHOD USED** | **ID** | **DG – JM – HT** | **SEX** | **GROUP -SEX** | **PC1**  **(SCORE)** | **PC2**  **(SCORE)** | **PC3**  **(SCORE)** |
| --- | --- | --- | --- | --- | --- | --- | --- | --- |
| 5  6  7  S2  S3  S4 | 1. Principal Component Analysis (PCA) (Fig. 5, 7, S2, S3, S4)  2. Visual Expression of Shape Variance along PC Axes using PCcontrib  (Fig. 6) | DG1 | Doigahama | Male | DM | -0.04346 | 0.000529 | -0.01086 |
|  |  | DG10 | Doigahama | Male | DM | -0.06998 | -0.00626 | 0.032188 |
|  |  | DG11 | Doigahama | Male | DM | -0.13961 | 0.046853 | 0.007904 |
|  |  | DG12 | Doigahama | Male | DM | -0.04227 | -0.03664 | 0.0386 |
|  |  | DG13 | Doigahama | Male | DM | -0.06779 | -0.00069 | 0.006077 |
|  |  | DG14 | Doigahama | Male | DM | -0.05407 | 0.034061 | 0.008394 |
|  |  | DG15 | Doigahama | Male | DM | 0.017916 | 0.005992 | -0.01173 |
|  |  | DG16 | Doigahama | Male | DM | -0.03613 | 0.031455 | -0.01664 |
|  |  | DG17 | Doigahama | Male | DM | -0.07218 | 0.053309 | -0.00878 |
|  |  | DG18 | Doigahama | Male | DM | 0.00229 | 0.000336 | -0.00307 |
|  |  | DG19 | Doigahama | Male | DM | 0.022265 | -0.04064 | -0.01045 |
|  |  | DG2 | Doigahama | Male | DM | -0.04339 | -0.02408 | 0.008162 |
|  |  | DG20 | Doigahama | Female | DF | -0.0128 | -0.0253 | 0.00039 |
|  |  | DG21 | Doigahama | Female | DF | -0.00357 | 0.001503 | 0.004633 |
|  |  | DG22 | Doigahama | Female | DF | 0.006934 | -0.00753 | -0.03816 |
|  |  | DG23 | Doigahama | Female | DF | 0.014935 | 0.009517 | 0.027209 |
|  |  | DG24 | Doigahama | Female | DF | -0.06959 | -0.01827 | 0.00132 |
|  |  | DG25 | Doigahama | Male | DM | 0.036593 | 0.017669 | -0.01105 |
|  |  | DG27 | Doigahama | Female | DF | -0.06479 | -0.0227 | 0.004186 |
|  |  | DG28 | Doigahama | Female | DF | -0.04943 | -0.00496 | -0.00248 |
|  |  | DG29 | Doigahama | Female | DF | -0.11756 | -0.00503 | -0.01869 |
|  |  | DG3 | Doigahama | Male | DM | 0.03015 | -0.00755 | -0.01688 |
|  |  | DG4 | Doigahama | Male | DM | 0.023142 | -0.00055 | -0.01531 |
|  |  | DG5 | Doigahama | Male | DM | -0.01511 | -0.01926 | -0.02033 |
|  |  | DG6 | Doigahama | Female | DF | -0.10733 | 0.003416 | 0.002293 |
|  |  | DG7 | Doigahama | Male | DM | -0.03858 | 0.09022 | 0.026844 |
|  |  | DG8 | Doigahama | Female | DF | -0.0828 | 0.024277 | 0.011133 |
|  |  | DG9 | Doigahama | Female | DF | -0.0653 | -0.02566 | 0.01682 |
|  |  | EN1 | Jomon | Female | JF | -0.03999 | -0.03794 | -0.00282 |
|  |  | GR1 | Jomon | Male | JM | -0.08271 | 0.026091 | -0.02096 |
|  |  | GR2 | Jomon | Male | JM | -0.00468 | 0.008646 | -0.02912 |
|  |  | HT1 | Hirota | Male | HM | 0.143865 | 0.005028 | 0.009049 |
|  |  | HT10 | Hirota | Male | HM | 0.049916 | -0.0064 | 0.006389 |
|  |  | HT11 | Hirota | Male | HM | 0.179554 | 0.025484 | 0.006919 |
|  |  | HT12 | Hirota | Male | HM | -0.01445 | -0.01365 | 0.033673 |
|  |  | HT13 | Hirota | Male | HM | 0.054494 | -0.00301 | 0.014293 |
|  |  | HT14 | Hirota | Male | HM | 0.058106 | 0.037647 | -0.07116 |
|  |  | HT15 | Hirota | Male | HM | 0.123922 | 0.026543 | 0.015988 |
|  |  | HT16 | Hirota | Female | HF | 0.085437 | -0.00869 | 0.034264 |
|  |  | HT17 | Hirota | Male | HM | -0.06314 | -0.00292 | 0.040969 |
|  |  | HT18 | Hirota | Female | HF | 0.102173 | 0.00536 | -0.00599 |
|  |  | HT19 | Hirota | Female | HF | 0.083002 | 0.01748 | -0.03104 |
|  |  | HT2 | Hirota | Male | HM | 0.126212 | 0.007145 | -0.00507 |
|  |  | HT3 | Hirota | Female | HF | 0.194071 | -0.03255 | 0.030776 |
|  |  | HT4 | Hirota | Male | HM | 0.089498 | -0.02117 | 0.024511 |
|  |  | HT5 | Hirota | Male | HM | 0.081361 | 0.044726 | 0.005982 |
|  |  | HT6 | Hirota | Male | HM | 0.085849 | -0.00049 | 0.012473 |
|  |  | HT7 | Hirota | Female | HF | -0.08809 | 0.004855 | -0.0016 |
|  |  | HT8 | Hirota | Female | HF | -0.02864 | 0.027534 | 0.043576 |
|  |  | HT9 | Hirota | Female | HF | 0.113239 | -0.01226 | 0.001051 |
|  |  | YM1 | Jomon | Female | JF | -0.10721 | -0.0201 | 0.002118 |
|  |  | YM2 | Jomon | Female | JF | -0.05216 | -0.03041 | -0.01212 |
|  |  | YM3 | Jomon | Male | JM | 0.016037 | 0.00487 | -0.05437 |
|  |  | YM4 | Jomon | Male | JM | -0.0973 | -0.0407 | -0.01001 |
|  |  | YM5 | Jomon | Male | JM | 0.040494 | -0.05703 | -0.04112 |
|  |  | YM6 | Jomon | Male | JM | -0.00733 | -0.02811 | -0.00839 |
|  |  |  |  |  |  |  |  |  |
| **Fig #** | **STAT.**  **METHOD USED** | **GROUP** | **PC** | **MIN** | **Q1** | **MEDIAN** | **Q3** | **MAX** |
| 8A | Boxplots of PC Scores (Doigahama, Jomon, Hirota)  (see associated PC scores above for base data) | Doigahama | 1 | -0.13961 | -0.06824 | -0.04283 | 0.003451 | 0.036593 |
|  |  | Doigahama | 2 | -0.04064 | -0.01852 | -0.00062 | 0.011555 | 0.09022 |
|  |  | Jomon | 1 | -0.10721 | -0.08271 | -0.03999 | -0.00468 | 0.040494 |
|  |  | Jomon | 2 | -0.05703 | -0.03794 | -0.02811 | 0.00487 | 0.026091 |
|  |  | Hirota | 1 | -0.08809 | 0.052205 | 0.085437 | 0.118581 | 0.194071 |
|  |  | Hirota | 2 | -0.03255 | -0.00755 | 0.004855 | 0.021482 | 0.044726 |
| 8B | Boxplots of PC Scores (Group & Sex)  (see associated PC scores above for base data) | Doigahama Female | 1 | -0.11756 | -0.07619 | -0.06479 | -0.00819 | 0.014935 |
|  |  | Doigahama Male | 1 | -0.13961 | -0.05407 | -0.03858 | 0.017916 | 0.036593 |
|  |  | Jomon Female | 1 | -0.08809 | 0.027179 | 0.085437 | 0.107706 | 0.194071 |
|  |  | Jomon Male | 1 | -0.06314 | 0.053349 | 0.083605 | 0.124495 | 0.179554 |
|  |  | Hirota Female | 1 | -0.10721 | -0.07968 | -0.05216 | -0.04607 | -0.03999 |
|  |  | Hirota Male | 1 | -0.0973 | -0.06386 | -0.00601 | 0.010857 | 0.040494 |
|  |  | Doigahama Female | 2 | -0.02566 | -0.02049 | -0.00503 | 0.002459 | 0.024277 |
|  |  | Doigahama Male | 2 | -0.04064 | -0.00755 | 0.000336 | 0.031455 | 0.09022 |
|  |  | Jomon Female | 2 | -0.03255 | -0.01048 | 0.004855 | 0.01142 | 0.027534 |
|  |  | Jomon Male | 2 | -0.02117 | -0.00386 | 0.002269 | 0.025749 | 0.044726 |
|  |  | Hirota Female | 2 | -0.03794 | -0.03417 | -0.03041 | -0.02526 | -0.0201 |
|  |  | Hirota Male | 2 | -0.05703 | -0.03755 | -0.01162 | 0.007702 | 0.026091 |
