# Supplementary figures and images for: Investigating intentional cranial modification: A hybridized two-dimensional/three-dimensional study of the Hirota site, Tanegashima, Japan
Source: PLoS One. 2023 Aug 16;18(8):e0289219. doi: 10.1371/journal.pone.0289219 (PMC10431670; doi:10.1371/journal.pone.0289219)

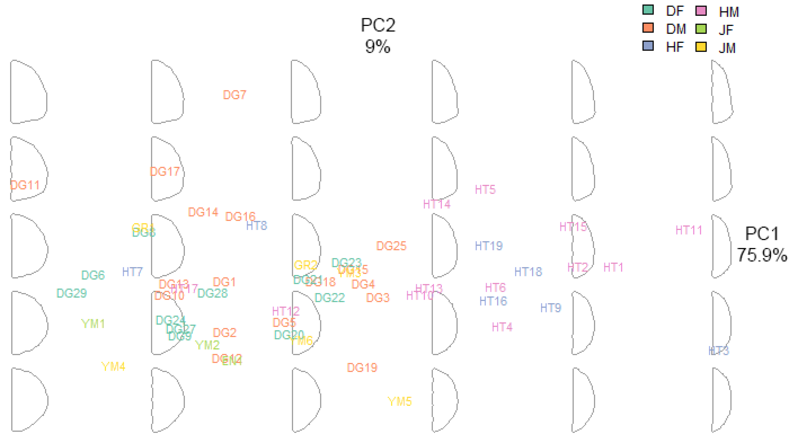

Supplement: S1 Fig — (TIF) [file pone.0289219.s003.tif]

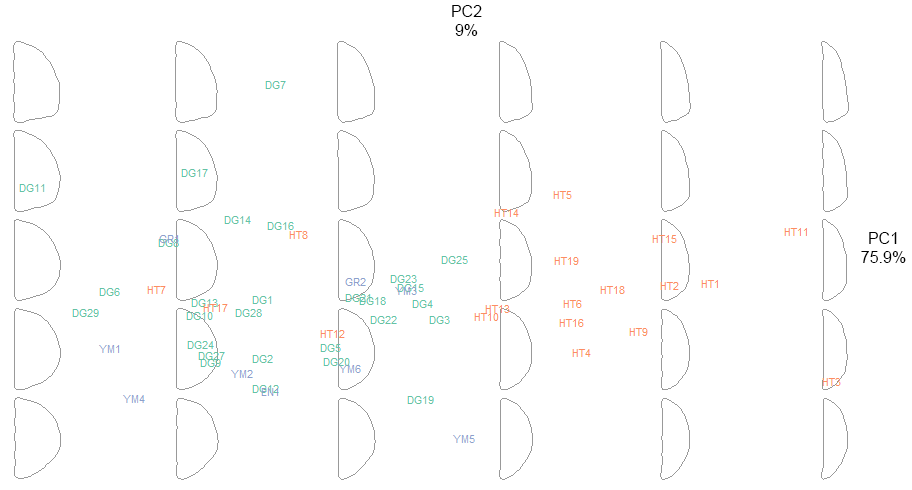

Supplement: S2 Fig — (TIF) [file pone.0289219.s004.tif]

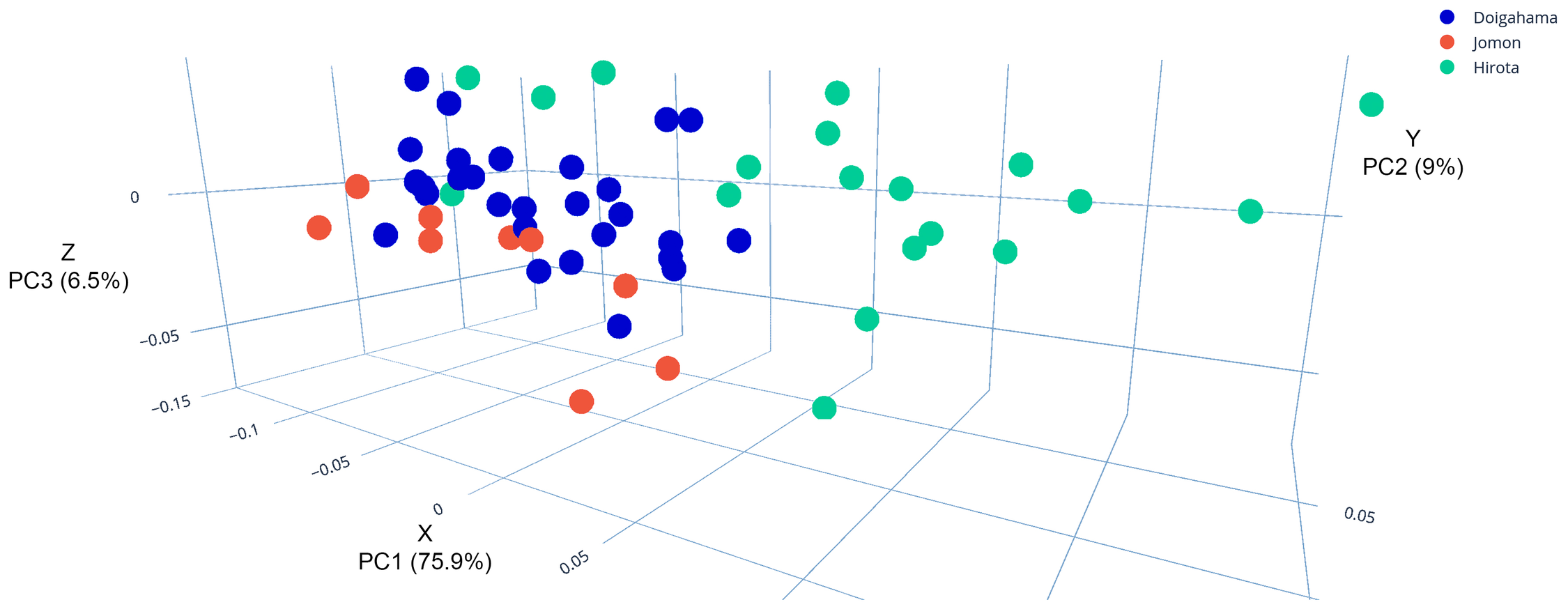

Supplement: S3 Fig — Colors denote site groupings: green: Hirota; blue: Doigahama; red: Jomon of Kyushu. PC1, PC2, and PC3 contribute 75.9%, 9.0%, and 6.5% of the variance, respectively. (TIF) [file pone.0289219.s005.tif]
